# Natural Products in Hashimoto’s Thyroiditis: Therapeutic and Thyroid-Disrupting Effects—A Critical Narrative Review

**DOI:** 10.3390/biomedicines14102273

**Published:** 2026-10-08

**Authors:** Dorota Szydlarska, Marta Zuzanna Ciechomska, Zuzanna Banaszkiewicz, Marek Brytan, Justyna Chałdaś-Majdańska, Melania Brzozowska, Andrzej Śliwczyński

**Affiliations:** 1 Department of Family Medicine, National Medical Institute of the Ministry of the Interior and Administration, Wołoska 137, 02-507 Warsaw, Poland; dorota.szydlarska@pimmswia.gov.pl (D.S.); marta.ciechomska@pimmswia.gov.pl (M.Z.C.); 2 Department of Family Medicine, Medical University of Warsaw, Binieckiego 6, 02-097 Warsaw, Poland; 3 Military Institute of Hygiene and Epidemiology, Kozielska 4, 01-163 Warsaw, Poland; marek.brytan@wihe.pl; 4 Faculty of Health Sciences, Warsaw Branch of WSEI University, Twarda 14/16, 00-824 Warsaw, Poland; justyna.chaldas-majdanska@wsei.pl (J.C.-M.);; 5 Central Office, National Health Fund, Rakowiecka 26/30, 02-528 Warsaw, Poland

**Keywords:** Hashimoto’s thyroiditis, autoimmune thyroiditis, natural products, phytochemicals, polyphenols, thyroid peroxidase, sodium–iodide symporter, immunomodulation

## Abstract

Hashimoto’s thyroiditis (HT) is a chronic autoimmune thyroid disease in which natural product-derived compounds may influence both immune-mediated injury and thyroid-specific physiology. This critical narrative review integrated molecular, cellular, animal, and human evidence using a dual-axis framework: H1, covering anti-inflammatory, immunomodulatory, antioxidant, and cytoprotective effects; and H2, covering direct effects on thyroid peroxidase, sodium–iodide symporter activity, thyroglobulin, thyroid-specific gene expression, and hormone synthesis. PubMed/MEDLINE, Scopus, and Web of Science searches identified 687 records; after deduplication, 356 unique publications were screened and 79 were retained for final synthesis. Human evidence was limited to three independent HT trials evaluating curcumin, Nigella sativa, and genistein. These studies reported preliminary immunological and/or biochemical thyroid-function signals, including changes in thyroid autoantibodies, inflammatory markers, TSH, or thyroid hormones. Reductions in TPOAb or TgAb are surrogate immunological outcomes and should not be interpreted as evidence of disease modification. None of the available trials established preservation of thyroid functional reserve, prevention or delay of hypothyroidism, reduced levothyroxine requirements, or improvement in patient-important outcomes. Preclinical studies supported biological plausibility but also identified direct thyroid modulation whose clinical relevance depends on exposure and context. Natural products should currently be regarded as investigational adjuncts rather than established disease-modifying therapies.

## 1. Introduction

Hashimoto’s thyroiditis (HT) is a chronic organ-specific autoimmune disorder in which loss of immune tolerance to thyroid antigens is accompanied by lymphocytic infiltration, inflammatory signaling, progressive injury of thyroid follicular cells, and, in many patients, eventual hypothyroidism. HT is a major cause of primary hypothyroidism in iodine-sufficient settings; recent global syntheses estimate its prevalence at approximately 5–10%, with women affected about four times as often as men [1,2]. Clinical management is highly effective when thyroid hormone deficiency is present: levothyroxine restores circulating hormone availability and remains the standard treatment of hypothyroidism. Nevertheless, hormone replacement is not a disease-modifying immunotherapy. It does not directly target the upstream autoimmune process, oxidative imbalance, inflammatory networks, or the immune-cell interactions that contribute to thyroid tissue injury. This distinction has stimulated interest in adjunctive approaches that might influence mechanisms operating before or alongside irreversible loss of thyroid function.

Natural product-derived bioactive compounds are particularly relevant to this question because many exert antioxidant, anti-inflammatory, immunomodulatory, or cytoprotective effects. Polyphenols, flavonoids, quinones, phytosterols, and complex botanical preparations can influence intracellular pathways implicated in autoimmune inflammation, including NF-κB, JAK/STAT signaling, Nrf2-regulated redox responses, inflammasome activity, cytokine production, and T-cell polarization. Clinical and experimental studies have reported potential benefits for several classes of polyphenols, flavonoids and botanical preparations. The human evidence remains limited, but randomized studies with Nigella sativa and, more recently, curcumin-containing interventions illustrate that clinically measurable outcomes such as thyroid autoantibodies, thyroid-stimulating hormone, inflammatory markers, and metabolic variables can be studied prospectively in patients with HT [3,4]. Experimental autoimmune thyroiditis models further support the biological plausibility of immunomodulatory effects; for example, luteolin has been shown to attenuate thyroid inflammation and STAT3-related signaling in mice [5].

A simple ‘antioxidant equals beneficial’ interpretation, however, is insufficient for thyroid disease. The thyroid gland is uniquely dependent on tightly regulated oxidative chemistry for hormone synthesis, and natural compounds capable of modifying redox signaling may also interact directly with thyroid-specific molecular processes. Experimental data show that some phytochemicals can alter thyroid peroxidase (TPO) activity, sodium–iodide symporter (NIS) expression or iodide uptake, thyroglobulin expression, thyroid-stimulating hormone receptor signaling, transcriptional control of thyroid-specific genes, or thyroid hormone synthesis.

Several natural compounds have demonstrated direct thyroid-disrupting effects despite possessing antioxidant or anti-inflammatory properties [6,7]. These observations support a dual-axis model for interpreting natural products in HT. The first axis concerns immunomodulation and cytoprotection: reduction in oxidative stress, inflammatory signaling, cytokine activity, pathological immune-cell polarization, and thyrocyte injury. The second concerns direct thyroid physiology: stimulation, inhibition, or other modulation of TPO, NIS/iodide transport, thyroglobulin, TSHR-related signaling, thyroid-specific transcription factors, and hormone synthesis. A compound may therefore have apparently favorable effects on one axis and unfavorable or dose-dependent effects on the other. The clinically relevant outcome is the net effect produced by the interaction of these two layers, further modified by dose, formulation, bioavailability, iodine status, baseline thyroid function, disease stage, and concomitant levothyroxine or other therapy.

The existing literature is heterogeneous in design and translational distance. It includes mechanistic assays, thyroid cell models, experimental autoimmune thyroiditis, other animal models of thyroid dysfunction, observational human data, and a relatively small number of interventional studies in patients with HT. These evidence types should not be treated as equivalent. A compound that improves an inflammatory pathway in vitro is not thereby established as an effective treatment for HT, and a change in a thyroid-specific molecular target may represent either a therapeutic opportunity or a safety signal depending on the clinical context. The resulting evidence base is therefore better suited to critical, mechanism-informed synthesis than to an undifferentiated catalogue of supplements.

The aim of this critical narrative review is to examine the potential therapeutic and thyroid-disrupting effects of defined natural product-derived bioactive compounds in Hashimoto’s thyroiditis by integrating molecular, cellular, animal, and human evidence. Particular emphasis is placed on the relationship between immune/redox effects and direct thyroid effects, the hierarchy of evidence supporting individual compounds, and the barriers that currently limit translation into routine clinical practice. The review is intended to identify not only promising adjunctive strategies but also situations in which apparently beneficial natural products may require caution because of direct effects on thyroid physiology.

## 2. Review Scope, Literature Search, and Evidence Framework

### 2.1. Review Scope and Interpretive Framework

This work was designed as a critical narrative review with a structured and transparent literature search and evidence selection process. It was not conceived as a systematic review and does not claim exhaustive identification of every potentially eligible publication. Methodological reporting was guided by the principles of the SANRA (Scale for the Assessment of Narrative Review Articles) framework, particularly explicit justification of the review, a clearly defined objective, description of the literature search, appropriate referencing, and critical rather than purely descriptive synthesis [8]. The predefined interpretive framework required simultaneous consideration of therapeutic/immunomodulatory effects and direct thyroid effects. Although the review incorporates structured searching, screening, and evidence classification procedures, it was designed as a critical narrative review intended to support mechanism-based synthesis rather than a systematic review aimed at exhaustive evidence capture.

### 2.2. Information Sources and Search Date

The primary bibliographic searches were conducted in PubMed/MEDLINE, Scopus, and Web of Science. The project search was completed on 24 August 2026. ClinicalTrials.gov was considered a supplementary source for identifying or cross-checking registered human interventional studies when a registry record was relevant to an included clinical publication. Database exports were retained in the project archive together with identifiers needed for cross-database matching and deduplication.

### 2.3. Search Architecture

Two complementary search axes were used. The H1 axis was designed to identify evidence linking Hashimoto’s thyroiditis or autoimmune thyroiditis with natural products through mechanisms relevant to autoimmunity, oxidative stress, inflammation, immunomodulation, cellular injury, and related signaling pathways. The H2 axis was deliberately broader and was designed to capture direct effects of natural products on thyroid follicular-cell physiology and thyroid-specific molecular targets, including TPO, NIS and iodide uptake, thyroglobulin, TSHR-related signaling, thyroid-specific gene expression, and thyroid hormone synthesis or secretion. This second axis was necessary to identify potential thyroid-disrupting or goitrogenic actions that would be missed by a search restricted to therapeutic studies in HT.

Search concepts combined disease terms such as ‘Hashimoto thyroiditis’, ‘autoimmune thyroiditis’, and ‘autoimmune hypothyroidism’ with intervention terms including ‘natural product’, phytochemical, polyphenol, flavonoid, alkaloid, and names of individual compounds or products. Compound-specific mechanistic searches were supplemented with thyroid-related terms such as thyroid peroxidase/TPO, sodium–iodide symporter/NIS, iodide uptake, thyroglobulin, TSH receptor, oxidative stress, autoimmunity, Th17/Treg, Tfh/Tfr, and cytokines. The complete database-specific search strategies used in PubMed/MEDLINE, Scopus, and Web of Science are reproduced in Supplementary Table S1. The database-specific search yields are summarized in Table 1. Search results and database exports were archived at the time of the searches.

### 2.4. Eligibility Criteria

Primary research was considered eligible when it addressed a defined natural product, botanical preparation, or identifiable bioactive compound and met at least one of two relevance conditions: (i) direct relevance to HT/autoimmune thyroiditis, including immune, inflammatory, oxidative, histological, serological, hormonal, or clinical outcomes; or (ii) direct relevance to thyroid follicular-cell function or a thyroid-specific molecular target that could materially inform benefit–risk interpretation in HT. Eligible evidence therefore included human clinical studies, studies using cells or immune cells obtained from patients with HT, human thyrocyte experiments, experimental autoimmune thyroiditis models, other animal models with direct thyroid endpoints, and mechanistic thyroid-cell or biochemical studies. Evidence from non-HT thyroid models was retained only when it directly informed the H2 safety/thyroid-function axis.

Reviews, systematic reviews, narrative reviews, and relevant chapters were used as background sources and for citation chasing but were not treated as primary evidence of efficacy. Records were excluded when the relationship to HT, autoimmune thyroiditis, or thyroid-specific physiology was too indirect; when the natural-product component was absent or could not be meaningfully separated from an unrelated intervention; when the study concerned unrelated disease mechanisms without thyroid relevance; when cancer-only findings did not provide direct information on a thyroid-specific target applicable to the review question; or when the publication type did not contribute primary evidence or useful contextual synthesis.

### 2.5. Record Management and Deduplication

Records exported from the three databases were merged into a common screening register. Deduplication used available bibliographic identifiers, including PMID, DOI, Scopus EID, and Web of Science accession number, supplemented by normalized title matching when identifiers were absent or inconsistent. The combined PubMed, Scopus, and Web of Science workflow yielded 356 unique records for screening. Multiple publications arising from the same clinical trial or experimental program were retained as separate publications for endpoint extraction but were flagged as a shared study cluster so that one underlying cohort would not be interpreted as independent evidence more than once.

### 2.6. Screening and Adjudication

Title and abstract screening was organized as an independent multi-reviewer process. Two experienced clinical reviewers with expertise in endocrinology independently classified each record as include, exclude, or uncertain. Reviewers performed the initial assessment independently and were blinded to each other’s classifications during the screening phase.

A third reviewer subsequently reassessed the screening register, with particular attention to records requiring adjudication, studies with uncertain eligibility, and methodological credibility. Final classifications were consolidated across the three reviewer assessments. Discrepant or uncertain decisions were resolved through reassessment and consensus discussion, while the original reviewer judgments were retained in the screening register for traceability. The earlier preliminary machine-assisted categorization used to organize the database was treated solely as a working aid and did not replace human review at any stage of the selection process.

After completion of the third-reviewer adjudication, 79 publications were retained in the final evidence set, 124 were retained as uncertain/background material, and 153 were excluded. Publications arising from the same clinical trial or experimental program were treated as linked study clusters when interpreting the strength of evidence. Secondary reviews retained for triangulation and citation chasing were not considered independent primary evidence of efficacy. Records remaining in the uncertain/background category after adjudication were not part of the 79-publication final evidence set and did not contribute as primary evidence to the principal synthesis. They were retained in the screening archive for traceability and could be consulted only for contextual background or citation chasing where appropriate.

### 2.7. Evidence Hierarchy and Data Extraction

Evidence was prioritized according to both methodological strength and proximity to the clinical question. Human interventional studies conducted directly in patients with HT were assigned the greatest translational weight, followed by other human studies and experiments using patient-derived cells or human thyrocytes. Experimental autoimmune thyroiditis models were considered particularly informative for immunological mechanisms, whereas other thyroid animal and cell models were used chiefly to characterize direct thyroid actions and safety signals. Mechanistic or biochemical studies were used to support pathway interpretation but not to infer clinical efficacy in isolation.

For publications selected for synthesis, extracted fields included: natural source and defined compound or preparation; formulation and dose; experimental model or patient population; HT diagnostic context and baseline thyroid functional state; concomitant levothyroxine or other relevant therapy; duration of exposure; immune and inflammatory endpoints; oxidative-stress endpoints; TPOAb and TgAb; TSH, total T3, total T4, fT3 and fT4 measures; thyroid histology or structural outcomes; TPO enzyme activity; NIS expression and iodide uptake; thyroglobulin and TSHR-related outcomes; thyroid-specific transcriptional effects; reported adverse or goitrogenic signals; and limitations relevant to translation, including bioavailability and product standardization.

Before final synthesis, evidence-type and evidence-level labels were manually audited against the study title, abstract, and available methodological description. Twelve records required correction of the evidence-type label and ten required correction of the evidence-level label. The corrected classifications were used in the evidence tables and narrative interpretation; original screening labels were retained in the project register for traceability. Classification correction was performed separately from the include/background/exclude adjudication. These corrections did not alter study eligibility, screening status, or the composition of the final evidence set. They did, however, change the evidentiary weighting assigned to affected records and were incorporated into the final evidence hierarchy and narrative interpretation. Original and corrected labels for affected records are documented in Supplementary Table S2.

Evidence-role classification. For the final synthesis, retained records were assigned an interpretive role. Core HT/AIT evidence comprised primary studies directly investigating Hashimoto’s thyroiditis or experimental autoimmune thyroiditis and reporting thyroid-relevant immunological, structural, functional, or mechanistic outcomes. Supportive thyroid-physiology evidence comprised primary studies outside HT/AIT that directly examined thyroid-specific processes relevant to the H2 axis, including TPO activity, NIS expression or iodide transport, thyroglobulin regulation, thyroid-specific gene expression, or thyroid hormone synthesis and function. Contextual evidence comprised the secondary literature and primary studies providing indirect mechanistic, metabolic, pharmacological, toxicological, pharmacokinetic, or exposure-related information relevant to interpretation but not constituting direct evidence of efficacy in HT. Contextual evidence was not treated as independent evidence of therapeutic efficacy. For coding purposes, circulating TPOAb and TgAb were treated as autoimmune/immunological outcomes on the H1 axis, whereas H2:TPO was reserved for direct effects on TPO enzyme activity/expression and H2:thyroglobulin for direct effects on thyroglobulin expression or protein. Autoantibody changes were therefore not interpreted as direct thyroid-physiology effects.

### 2.8. Narrative Synthesis and Interpretation

Because of substantial heterogeneity in compounds, formulations, doses, experimental models, populations, outcomes, and study design, no meta-analysis was planned. Evidence was synthesized narratively by compound and by mechanistic domain. For each compound, findings were mapped onto two parallel axes: (A) immunomodulatory/cytoprotective effects, including oxidative stress, inflammatory signaling, cytokine networks, immune-cell balance, and thyrocyte injury; and (B) direct thyroid effects, including TPO, NIS/iodide uptake, thyroglobulin, TSHR-related signaling, thyroid-specific gene expression, and hormone synthesis. The final interpretation emphasized the net clinical effect rather than assuming that an antioxidant or anti-inflammatory effect necessarily predicts benefit in HT.

Clinical translation was evaluated with explicit attention to differences between purified compounds and whole botanical products, dose and formulation, bioavailability, disease stage, baseline euthyroid versus hypothyroid state, iodine status, concomitant therapy, and the distinction between mechanistic plausibility and patient-important outcomes. Conflicting or bidirectional findings were retained rather than harmonized, because the possibility of simultaneous therapeutic and thyroid-disrupting effects is a central hypothesis of this review.

For the revised compound-level synthesis, H1 and H2 evidence was considered separately and then compared within each compound or defined preparation. Absence of evidence on either axis was classified as insufficient evidence rather than neutral activity. Discordant findings across experimental models, exposure conditions, or evidence levels were retained explicitly and were not collapsed into a single favorable or unfavorable classification. Interpretation also considered whether evidence originated from patients with HT, autoimmune thyroiditis models, other thyroid models, or mechanistic systems outside HT.

### 2.9. Generative AI Assistance

Generative artificial intelligence (OpenAI ChatGPT, GPT-5.6 Sol; OpenAI, San Francisco, CA, USA; September 2026) was used during manuscript preparation to support drafting and refinement of database search strings, organization of the extracted literature, preliminary machine-assisted categorization, translation, text condensation and structural organization, editorial assistance, and synthesis of the literature summaries supplied by the authors. All database searches were executed by the authors. Eligibility decisions, reviewer adjudication, evidence classification, and clinical interpretation were performed by human reviewers; AI-assisted outputs did not replace human screening or decision-making. The authors independently verified all scientific claims, numerical results, and bibliographic references, reviewed and edited all AI-assisted content, and took full responsibility for the final manuscript. The prompt history for substantive AI-assisted tasks is retained in the project chat archive and can be compiled and provided to the journal upon request.

### 2.10. Ethical Considerations

The review uses only previously published or publicly registered research and does not involve recruitment of human participants, intervention in patient care, or analysis of identifiable individual-level patient data. Institutional review board or research ethics committee approval was therefore not required for the review itself.

## 3. Results

### 3.1. Search Results and Publication Selection

The literature search was organized around two complementary thematic axes. H1 targeted potential anti-inflammatory, immunomodulatory, antioxidant, and cytoprotective actions of natural products and bioactive compounds in the context of autoimmune thyroiditis. H2 addressed direct effects on thyroid physiology, particularly thyroid peroxidase (TPO), thyroglobulin, sodium-iodide symporter (NIS)-dependent iodide uptake, TSH receptor-related signaling, and thyroid hormone synthesis or metabolism.

Across the six database-axis combinations, the searches yielded 687 database records: 145 from PubMed/MEDLINE, 337 from Scopus, and 205 from Web of Science. After merging H1 and H2 exports and removing within- and cross-database duplicates, 356 unique publications remained for screening, corresponding to 331 duplicate records removed.

At the preliminary classification stage, 73 publications were marked as include, 121 as uncertain/background, and 162 as exclude. These preliminary labels were subsequently reassessed during third-reviewer adjudication and consolidation of the reviewer assessments. Following this process, 79 publications (22.2%) were retained in the final evidence set, 124 (34.8%) were retained as uncertain/background material, and 153 (43.0%) were excluded. The preliminary screening labels were therefore not treated as the final evidence-role classification; retained studies were subsequently assigned a role as core HT/AIT evidence, supportive thyroid-physiology evidence, or contextual evidence. The 124 uncertain/background records were outside the final 79-publication evidence set and were not included in the core/supportive evidence synthesis or the quantitative characterization of retained evidence; they remained available only as archived contextual material or citation-chasing leads.

**Figure 1:**
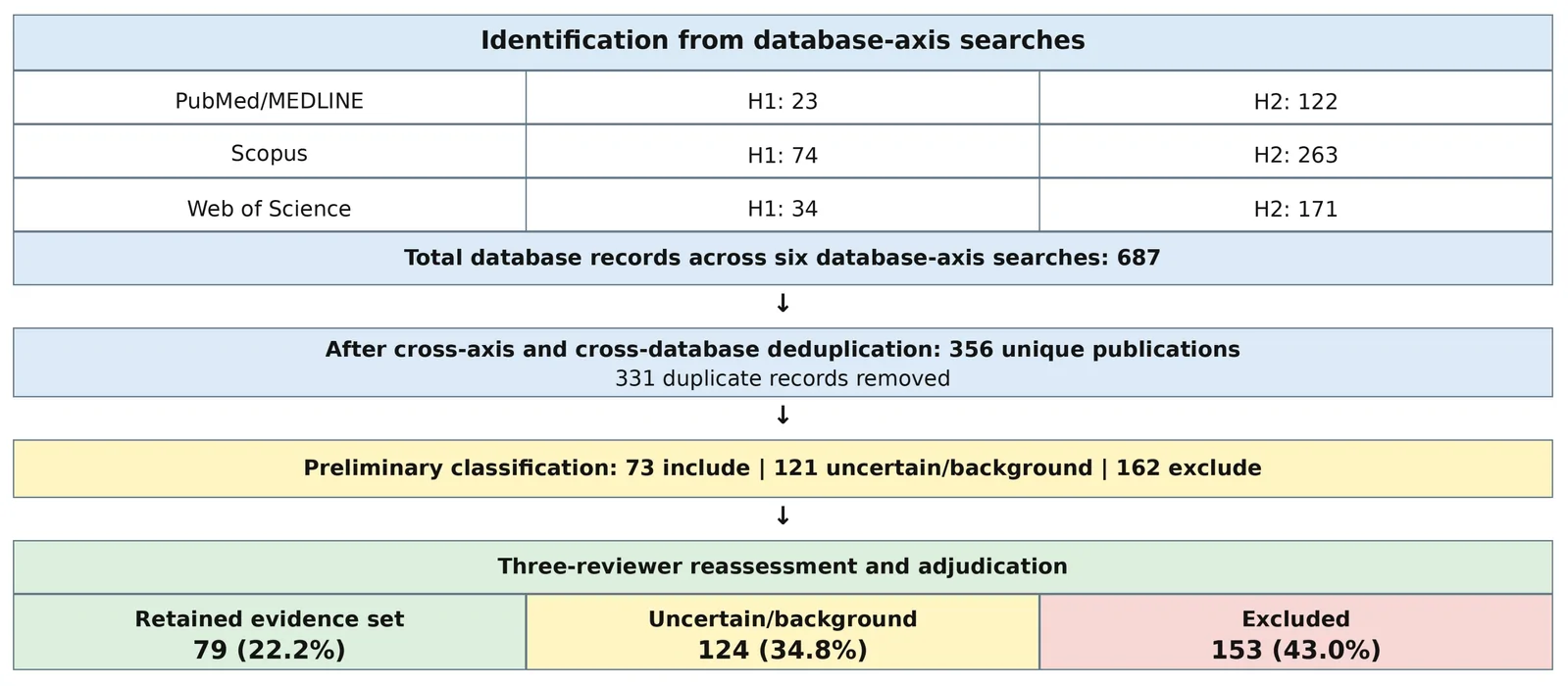
Structured literature search and evidence selection flow for the critical narrative review. Abbreviations: H1, immune/redox axis; H2, direct thyroid-physiology axis.

Among the 79 retained publications, 58 (73.4%) were identified exclusively through H2, 8 (10.1%) exclusively through H1, and 13 (16.5%) through both search axes. Thus, 71 of 79 publications (89.9%) contained a component directly relevant to thyroid function, whereas 21 (26.6%) addressed inflammatory, immunomodulatory, or antioxidant mechanisms; these categories were not mutually exclusive. Cross-database overlap was substantial: 74 retained publications were indexed in Scopus, 66 in Web of Science, and 57 in PubMed/MEDLINE, with 56 represented in all three databases. The evidence selection process is summarized in Figure 1.

### 3.2. General Characteristics of the Evidence Base

The retained publications were published between 1991 and 2026. The evidence base became markedly denser in the most recent decade: 48 of 79 publications (60.8%) were published from 2015 onward and 30 (38.0%) from 2020 onward.

Preclinical evidence predominated and included animal models, thyroid-cell systems, human thyrocytes, patient-derived immune cells, and biochemical or enzyme-based mechanistic assays. Human interventional evidence was considerably smaller. Two systematic reviews were retained for triangulation of the primary evidence, together with selected secondary syntheses used for contextual interpretation rather than as independent evidence of efficacy [9,10].

Flavonoids and other polyphenols were the most frequently represented compound classes. The most recurrent individual compounds or products were genistein (18 records), quercetin (11), daidzein and catechins (8 each), Nigella sativa (7), luteolin (6), resveratrol, apigenin, and rutin (5 each), and curcumin (4). Other retained evidence concerned EGCG, naringenin, hesperidin, rosmarinic acid, morin, Prunella vulgaris-derived constituents, citrus polymethoxyflavones, Withania somnifera, and multicomponent botanical preparations. Individual publications could contribute to more than one compound or mechanistic category.

The final evidence-role audit further distinguished the broad 79-publication evidence set from a narrower core of primary studies directly addressing HT or experimental autoimmune thyroiditis (AIT). Following reassessment of outcome relevance, 18 publications constituted the core evidence set: six clinical reports representing three independent clinical trials, one ex vivo study using lymphocytes from patients with HT, and eleven preclinical or mechanistic HT/AIT studies. Primary studies conducted outside HT/AIT but directly interrogating thyroid-specific processes were classified as supportive thyroid-physiology evidence, whereas the secondary literature and studies providing indirect mechanistic, metabolic, pharmacological, toxicological, or exposure-related information were classified as contextual evidence. Table 2 summarizes the core primary evidence and explicitly identifies linked publications arising from the same clinical trial.

The study by Kayumov et al. [26], although conducted in an experimental AIT model, was reclassified as contextual metabolic evidence because its principal outcomes—pancreatic hormones, glucose homeostasis, HbA1c, and hepatic glycogen—did not directly assess thyroid autoimmunity or thyroid-specific physiology. The methodological characteristics of the three independent clinical trials are summarized in Table 3.

A compound-level integration of H1 and H2 evidence is presented in Table 4.

### 3.3. Anti-Inflammatory, Immunomodulatory, and Antioxidant Effects: H1 Axis

Within the retained evidence set, 39 publications were coded as addressing oxidative-stress or antioxidant mechanisms, 37 as addressing inflammation or immunomodulation, and 6 as addressing apoptosis or thyrocyte survival. These mechanistic domains frequently overlapped within individual studies.

The most direct clinical evidence in patients with HT was identified for curcumin, Nigella sativa, and genistein. In a randomized trial of 57 patients with HT that generated two publications reporting different outcome domains, addition of curcumin to an anti-inflammatory diet was associated with a significant adjusted between-group improvement in hs-CRP (*p* = 0.009). IL-6 decreased significantly within the curcumin group but did not show significant adjusted between-group superiority (*p* = 0.271). NF-κB decreased within the curcumin group and differed between groups at follow-up (*p* = 0.019), while the adjusted treatment effect was borderline (*p* = 0.051) [11]. The companion report found a significant between-group reduction in TPOAb (*p* = 0.006), while between-group superiority was not consistently demonstrated for TSH and total T3 after accounting for baseline and within-group changes [4].

Several publications on Nigella sativa derived from the same or closely linked HT clinical program and reported complementary endpoints. Eight-week supplementation with powdered black cumin seeds was associated with higher total antioxidant capacity and superoxide dismutase activity and lower malondialdehyde, together with reductions in VCAM-1 [13]. Related reports described reductions in IL-23, TSH, and TPOAb and an increase in total T3 [3,14]. Ex vivo experiments using lymphocytes from women with HT demonstrated marked effects of Nigella sativa essential oil on T-cell proliferation, cytokine production, and cell survival [12]. By contrast, a separate randomized trial in levothyroxine-treated patients with hypothyroidism did not demonstrate a clear improvement in thyroid function, underscoring heterogeneity across populations and formulations [27].

In a randomized placebo-controlled study of genistein in patients with HT, treatment was associated with higher total T4 and fT4 and lower TSH, TPOAb, and TgAb than placebo. Changes in Th1-related cytokines, including IFN-gamma and IL-2, supported an immunomodulatory component of the observed response [15]. As this evidence derives from a single clinical trial without independent replication, these findings should be interpreted as preliminary rather than confirmatory.

Preclinical studies also reported potentially favorable inflammatory or autoimmune effects for luteolin, morin, EGCG, Prunella vulgaris, citrus polymethoxyflavones, and multicomponent herbal preparations. Reported mechanisms included modulation of STAT3-related signaling, NLRP3/caspase-1, HMGB1/TLR9, IL-17, and NF-kB pathways; induction of IDO1/Treg-related responses; activation of Nrf2-linked antioxidant defense; and attenuation of inflammatory infiltration, apoptosis, and follicular injury in experimental autoimmune thyroiditis [5,17,20,21,22,23,25].

### 3.4. Direct Effects on Thyroid Physiology: H2 Axis

The larger evidence domain concerned direct actions of natural compounds on thyroid physiology. Thyroid hormone synthesis or functional thyroid endpoints were represented in 51 publications (64.6%), TPO-related effects in 39 (49.4%), thyroglobulin-related effects in 25 (31.6%), and NIS expression or iodide uptake in 17 (21.5%). TSHR-related signaling and deiodinase activity were evaluated less frequently.

The direction of these effects was not uniform. Several flavonoids and polyphenols inhibited TPO activity, thyroid-specific gene expression, or iodide uptake, whereas other compounds increased NIS expression or iodide uptake. In some cases, the same compound produced different or even opposite thyroid effects depending on experimental system, exposure duration, dose, and endpoint.

Soy isoflavones formed one of the largest mechanistic clusters. Genistein and daidzein inhibited TPO-catalyzed reactions in biochemical models, and dietary genistein inactivated rat TPO in vivo without producing an overt hypothyroid phenotype [28,29]. At the clinical level, a three-year randomized trial of genistein aglycone in postmenopausal women without HT found no significant adverse effect on thyroid function [30], whereas short-term high soy intake in healthy volunteers produced a transient TSH increase in men but not women [31]. These findings illustrate the translational gap between biochemical antithyroid activity and clinically detectable thyroid dysfunction.

Similar heterogeneity was observed for resveratrol, quercetin, rutin, and apigenin. Resveratrol and quercetin have been shown to suppress NIS or other thyroid-restricted genes and thyroid function in experimental systems [6,32,33], while rutin increased thyroid iodide uptake and NIS expression in rat and thyroid-cell models [34,35]. Apigenin enhanced TSH-stimulated radioiodide accumulation under defined experimental conditions [36]. These data support compound-, context-, and time-dependent rather than class-wide interpretation of flavonoid effects on the thyroid.

A 2026 multi-assay screen of 424 natural compounds provided an especially informative cross-compound test of the dual-axis concept. Of 424 compounds, 69 activated an antioxidant-response-element reporter, but only a small subset showed a profile consistent with predominantly Nrf2-dependent activation. In parallel, the screen identified independent and only partially concordant effects on thyroglobulin promoter activity, NIS-related endpoints, and iodide uptake. Antioxidant activity therefore did not reliably predict the direction of thyroid-specific effects [37].

### 3.5. Integrated Pattern of Evidence

Integration of the H1 and H2 evidence revealed two partly independent dimensions of biological activity. The first comprised potentially favorable anti-inflammatory, antioxidant, immunomodulatory, and cytoprotective actions, with clinically or experimentally relevant signals for curcumin, Nigella sativa, genistein, luteolin, Prunella vulgaris, and selected other flavonoids [3,4,5,11,12,13,14,15,17,20,21,22,23,25,27]. The second comprised direct modulation of thyroid hormone biosynthesis, TPO, thyroglobulin, NIS-dependent iodide transport, and thyroid-specific gene expression [6,28,29,30,31,32,33,34,35,36,37].

Consequently, a compound with favorable effects on oxidative stress or immune signaling did not necessarily have a favorable direct effect on the thyrocyte. For several compounds, potentially beneficial immunomodulatory actions coexisted with inhibition of TPO, NIS, or other elements of thyroid hormone synthesis. Conversely, some compounds that increased iodide uptake or NIS expression did not have direct evidence of disease-modifying activity in HT.

The most direct human evidence in patients with HT was available for curcumin, Nigella sativa, and genistein, but the number of independent interventions remained limited [3,4,11,12,13,14,15,27]. Several publications reported different outcomes from the same clinical trial or study cluster; publication counts therefore should not be interpreted as counts of independent clinical experiments.

The two retained systematic reviews likewise emphasized heterogeneity in interventions, dose, duration, populations, and endpoints and the predominance of surrogate outcomes, including thyroid autoantibodies, TSH and thyroid hormone concentrations, and inflammatory or oxidative-stress markers [9,10].

Overall, the retained evidence demonstrates substantial biological activity of natural products in mechanisms relevant to HT, but also considerable heterogeneity in the direction and magnitude of their direct thyroid effects. The resulting pattern supports assessment of each intervention through the combined immune/redox and direct-thyroid axes rather than through an assumption that antioxidant or anti-inflammatory activity alone predicts clinical benefit.

**Figure 2:**
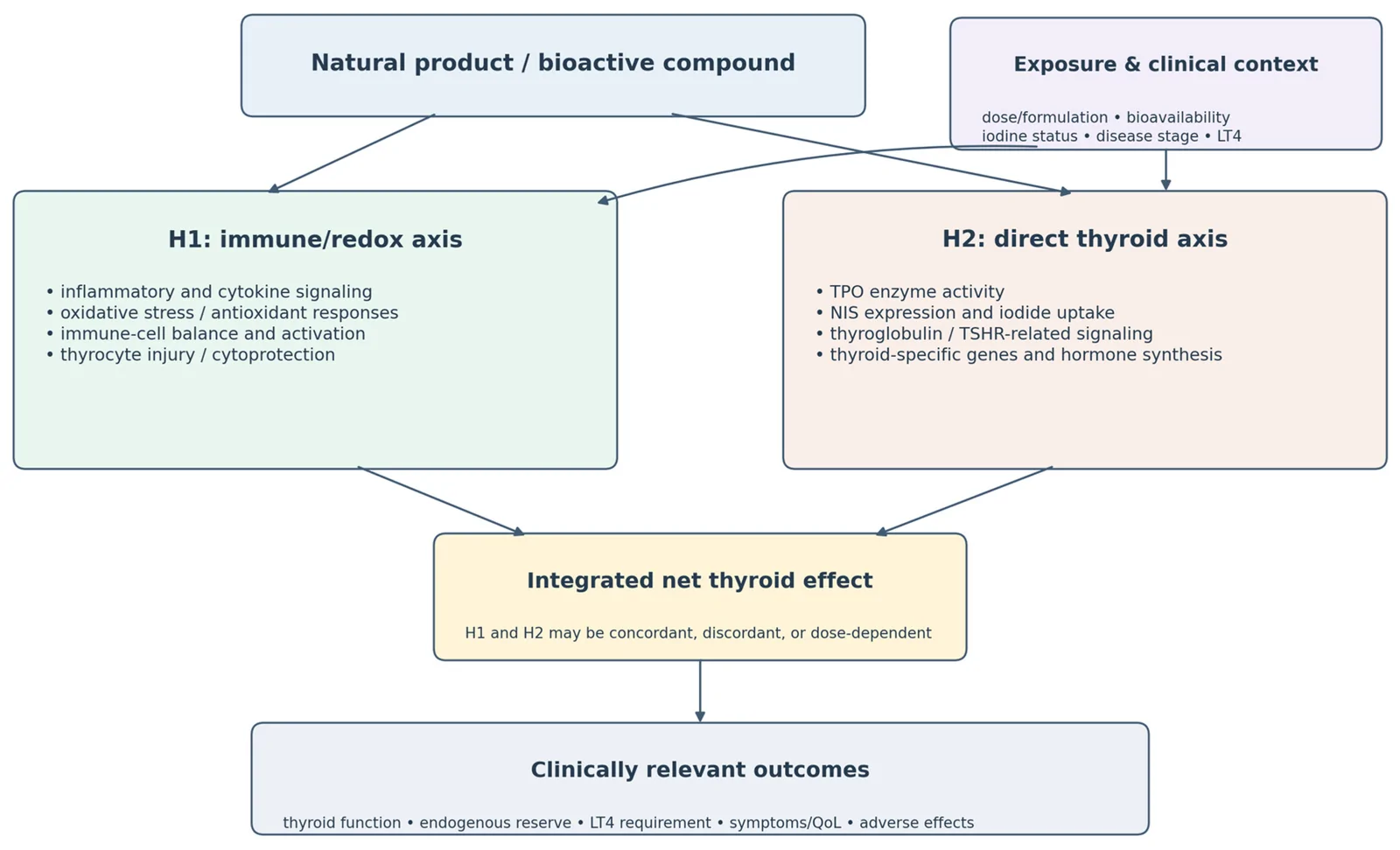
Proposed dual-axis framework for interpreting natural product-derived compounds in Hashimoto’s thyroiditis. H1 and H2 effects are considered separately and integrated in the context of dose, formulation, bioavailability, iodine status, baseline thyroid function, disease stage, and concomitant therapy to determine the net clinical effect.

Compound-level integration demonstrated that H1 and H2 are complementary but partly independent dimensions. Genistein provided the clearest cross-model discordance, with favorable immunological and biochemical findings in one HT trial but experimental TPO inhibition. Quercetin likewise combined potentially favorable inflammatory effects with inhibitory thyroid-specific actions. For resveratrol, rutin, and apigenin, evidence was predominantly mechanistic on the H2 axis; absence of corresponding HT evidence was therefore classified as insufficient evidence rather than neutrality. Increased NIS expression or iodide uptake was not interpreted as therapeutic efficacy, and isolated TPO/NIS inhibition was not interpreted as clinically demonstrated hypothyroidism. The proposed dual-axis framework and key modifiers of clinical translation are summarized in Figure 2.

## 4. Discussion

### 4.1. Why Do H1 and H2 Effects Diverge Across Models?

The principal finding of this review is not that natural products are uniformly beneficial or harmful in HT, but that immunological/redox activity and direct thyroid-specific activity represent partially independent dimensions of biological effect. Apparent discordance between H1 and H2 findings is therefore not necessarily contradictory. A compound may attenuate inflammatory signaling or oxidative stress while simultaneously modifying TPO activity, NIS expression, iodide transport, or thyroid-specific gene expression. Genistein illustrates this distinction particularly clearly: favorable immunological and biochemical findings have been reported in a clinical HT trial, whereas experimental systems demonstrate TPO-inhibitory activity. Quercetin similarly combines anti-inflammatory or antioxidant properties with experimental inhibition of thyroid-specific gene expression and iodide handling. Differences across studies may reflect model, dose, formulation, route, duration, iodine availability, and the presence or absence of autoimmune inflammation. Findings from isolated enzyme systems, cultured thyroid cells, experimental AIT, healthy animals, and patients with established HT therefore address different biological questions. The dual-axis framework prevents favorable activity on one axis from being assumed to predict a favorable net thyroid effect; absence of evidence on one axis is interpreted as insufficient evidence rather than biological neutrality.

### 4.2. Do Immunological Improvements Translate into Functional Thyroid Benefit?

The available clinical evidence does not establish that improvement in immunological markers translates into preservation of thyroid function. Only three independent clinical trials were identified, despite multiple clinical publications, and their findings predominantly concern immunological biomarkers and biochemical thyroid-function measures rather than patient-important or disease-modifying outcomes. Reductions in TPOAb or TgAb may indicate changes in immunological activity but do not independently demonstrate preservation of thyroid functional reserve, prevention or delay of hypothyroidism, reduced LT4 requirements, or symptomatic benefit. Similarly, short-term changes in TSH or circulating thyroid hormones do not establish modification of the natural history of HT. Methodological limitations further restrict inference. In the curcumin trial, both groups received an anti-inflammatory diet and the final analysis excluded participants exceeding the prespecified non-adherence threshold. The Nigella sativa evidence derives from one small 8-week trial reported across three publications rather than independent replications. In the genistein trial, 278 participants were randomized and 218 were included in the reported analysis, corresponding to 21.6% post-randomization attrition. The human evidence should therefore be interpreted as preliminary evidence of immunological and biochemical effects rather than disease-modifying efficacy.

### 4.3. Which Mechanistic Findings Are Exposure-Relevant?

A major translational uncertainty is whether experimental H2 effects occur at exposures relevant to human use. Demonstration of TPO inhibition, altered NIS expression, changes in thyroid-specific genes, or modified iodide uptake establishes biological activity but does not by itself demonstrate clinically relevant thyroid disruption or therapeutic efficacy. This distinction is particularly important for resveratrol, quercetin, rutin, and apigenin, for which key thyroid-specific observations originate predominantly from experimental systems. Interpretation requires consideration of concentration, dose, route of administration, duration, oral bioavailability, metabolism, circulating metabolites, protein binding, and thyroid tissue exposure. Experimental concentrations may not correspond to those achievable in the human thyroid after oral administration. Conversely, increased NIS expression or iodide uptake under defined experimental conditions should not be interpreted as a favorable HT effect without evidence of preserved thyroid function or clinically meaningful benefit. For several compounds, thyroid-specific pharmacokinetic data are insufficient to establish correspondence between experimental and clinically achievable tissue exposure. Differences between purified compounds and complex botanical preparations add further uncertainty. Accordingly, experimental H2 findings are treated as mechanistic signals unless exposure relevance and functional consequences are demonstrated in humans.

### 4.4. How Do Iodine Status and LT4 Treatment Modify Interpretation?

Clinical interpretation is further modified by iodine status and background LT4 therapy. Iodine availability influences thyroid hormone synthesis and may modify autoimmune thyroid processes; consequently, changes in TPO activity, NIS expression, or iodide uptake cannot be interpreted independently of iodine exposure. Increased iodide uptake is not intrinsically beneficial in HT, just as inhibition of an isolated component of hormone synthesis does not establish clinically relevant hypothyroidism. LT4 treatment introduces a separate interpretive problem. In treated patients, changes in TSH or circulating thyroid hormones may reflect residual endogenous thyroid function, treatment adherence, LT4 dose or exposure, or an intervention-related effect. Direct pharmacodynamic modulation of endogenous hormone synthesis should therefore be distinguished from pharmacokinetic interactions affecting LT4 absorption or treatment response. The included clinical trials did not characterize iodine exposure and background thyroid treatment with sufficient consistency to determine whether these factors modified intervention effects. Where LT4 use or treatment stability was reported, these characteristics should be considered explicitly; where they were not reported, the resulting uncertainty should not be treated as evidence that these factors were controlled.

### 4.5. What Would Constitute a Favorable Net Clinical Effect?

Within the proposed framework, a favorable net clinical effect requires more than activity on either H1 or H2 alone. A clinically useful intervention would ideally demonstrate a reproducible favorable effect on relevant autoimmune or inflammatory pathways without clinically important impairment of thyroid-specific physiology, at an exposure achievable with the intended formulation and route of administration. Most importantly, these biological effects would need to translate into outcomes relevant to patients. Future trials should therefore move beyond isolated changes in autoantibodies or short-term biochemical markers. Relevant outcomes include longitudinal preservation of endogenous thyroid function, progression to overt hypothyroidism, LT4 initiation or dose requirement, symptoms, quality of life, and adverse events. Trials should prospectively characterize baseline thyroid function, LT4 use and dose stability, iodine status or exposure, formulation and adherence, and should use adequate randomization, allocation concealment, prespecified outcomes, appropriate handling of missing data, and sufficiently long follow-up. Pharmacokinetic or exposure data would be particularly valuable for compounds for which experimental H2 activity raises efficacy or safety questions. The current evidence supports biological plausibility and hypothesis generation rather than routine therapeutic use.

### 4.6. Strengths and Limitations

This review has several strengths. The structured dual-axis search was designed to capture both potentially beneficial immune/redox effects and direct thyroid-specific actions that may otherwise be overlooked in reviews focused exclusively on supplementation efficacy. Multiple publications originating from the same clinical trial were identified as linked reports rather than treated as independent clinical evidence; negative and discordant findings were retained, and the revised synthesis explicitly distinguishes core HT/AIT evidence from supportive thyroid-physiology and contextual evidence. Several limitations remain. This was a structured critical narrative review rather than a systematic review and meta-analysis, and heterogeneity in interventions, models, exposures, and outcomes precluded quantitative synthesis. Human evidence was limited to three independent trials and predominantly surrogate outcomes. Much of the H2 evidence derives from biochemical, cellular, or animal systems for which correspondence with human thyroid tissue exposure is uncertain. Reporting of iodine status, background LT4 treatment, intervention adherence, pharmacokinetics, and patient-important outcomes was inconsistent. Formal risk-of-bias instruments were not applied uniformly across the heterogeneous study types. These limitations restrict causal and clinical inference and reinforce the need for independently replicated, adequately powered, longer-term human studies.

## 5. Conclusions and Research Priorities

### 5.1. Conclusions

Natural product-derived interventions in Hashimoto’s thyroiditis should be interpreted through two partly independent dimensions: immune/redox effects and direct thyroid-specific effects. Favorable H1 activity does not reliably predict favorable H2 activity; therefore, antioxidant or anti-inflammatory properties alone are insufficient to establish a favorable net clinical effect.

Human evidence is limited to three independent HT trials of curcumin, Nigella sativa, and genistein. These studies provide preliminary immunological and biochemical signals, but not evidence of durable disease modification, prevention of hypothyroidism, reduced LT4 requirements, or improvement in patient-important outcomes. The six principal clinical reports arise from only three trials, and small sample sizes, short follow-up, linked publications, and substantial attrition in the genistein trial further limit inference.

Preclinical and mechanistic studies support biological plausibility but also show potentially opposing thyroid-specific actions within the same compound. Modulation of TPO enzyme activity, NIS, iodide uptake, or thyroid-specific gene expression should be regarded as mechanistic thyroid activity rather than clinically demonstrated benefit or harm unless confirmed in humans at exposure-relevant doses.

Accordingly, natural products remain investigational adjuncts rather than substitutes for levothyroxine when hormone replacement is indicated. Demonstrating a favorable net effect will require standardized preparations, exposure-relevant mechanistic data, independent clinical replication, and outcomes that extend beyond surrogate laboratory markers.

### 5.2. Research Priorities

Research should prioritize independent, adequately powered randomized trials of interventions with direct HT data, particularly curcumin, Nigella sativa, and genistein. Trials should use standardized and chemically characterized preparations, prespecified dosing, sufficiently long follow-up, and stable background thyroid therapy where applicable.

Future studies should assess H1 and H2 domains in parallel and prospectively document baseline thyroid function, levothyroxine use and dose stability, iodine status or exposure, formulation, adherence, and adverse events. Patient-important outcomes—including symptoms, quality of life, progression to overt hypothyroidism, and levothyroxine initiation or dose requirement—should accompany autoantibodies and biochemical thyroid-function measures.

Compounds supported mainly by preclinical evidence require staged translational development, whereas compounds with documented TPO/NIS/iodide-uptake or thyroid-specific gene effects require explicit exposure-relevant thyroid-safety assessment. Future evidence syntheses should preserve study-cluster relationships, apply design-appropriate risk-of-bias assessment, and retain negative or discordant findings.

## Figures and Tables

**Table 1:** Search yields recorded in the project search log.

Database	H1 Records	H2 Records	Role in Review
PubMed/MEDLINE	23	122	Core biomedical search
Scopus	74	263	Broad multidisciplinary coverage
Web of Science	34	171	Cross-database validation and additional records

Abbreviations: H1, immune/redox axis; H2, direct thyroid-physiology axis.

**Table 2:** Core primary studies directly addressing Hashimoto’s thyroiditis or experimental autoimmune thyroiditis. (**A**) Human clinical and patient-derived evidence. (**B**) Core preclinical and mechanistic HT/AIT evidence.

(A)						
Study (Ref.)	Product/Intervention	Population/Model	Design/Exposure	Key Endpoints	Main Finding	Axis/Interpretive Role
Bourbour et al., 2026 [11]	Curcumin	57 patients with HT	Double-blind RCT; curcumin 1320 mg/day + anti-inflammatory diet vs. placebo + same diet; 12 wk	IL-6, NF-kB, hs-CRP	Adjusted hs-CRP improved versus placebo (*p* = 0.009). IL-6 decreased within the curcumin group without a significant adjusted between-group effect (*p* = 0.271). NF-κB differed between groups at follow-up (*p* = 0.019), while the adjusted treatment effect was borderline (*p* = 0.051).	H1; inflammatory signal. Same trial as [4] (NCT05975866).
Bourbour et al., 2026 [4]	Curcumin	57 patients with HT	Double-blind RCT; curcumin 1320 mg/day + anti-inflammatory diet vs. placebo + same diet; 12 wk	TPOAb, TSH, total T3/total T4, lipids	TPOAb decreased versus placebo; no consistent adjusted between-group superiority for TSH/total T3.	H1/H2; autoimmunity signal. Same trial as [11].
Ciesielska-Figlon et al., 2023 [12]	Nigella sativa essential oil	T lymphocytes from women with HT and healthy controls	Ex vivo; N. sativa essential oil 0.00025–0.025%; endpoints assessed at 72 and 120 h	CD4/CD8 proliferation, cytokines, apoptosis	Strong modulation of T-cell proliferation/cytokines; higher exposure also increased cell death.	H1; patient-derived mechanistic evidence and concentration-dependent safety signal.
Abbasalizad Farhangi & Tajmiri, 2020 [13]	Nigella sativa	40 patients with HT	Randomized placebo-controlled trial; powdered N. sativa seeds 2 g/day; 8 wk	TAC, SOD, GPX, MDA, ICAM-1, VCAM-1	Improved selected oxidative-stress markers and VCAM-1; metabolic/anthropometric changes also reported.	H1; same trial cluster as [3,14] (IRCT2015021719082N4).
Zhang et al., 2017 [15]	Genistein	218 patients with HT	Double-blind placebo-controlled RCT; genistein 600 mg/day (purified soy extract); 1 mo	total T3, total T4, fT4, TSH, TPOAb, TgAb, IFN-gamma, IL-2	Higher total T4/fT4 and lower TSH/TPOAb/TgAb; Th1-cytokine changes supported immunomodulation.	H1/H2; independent clinical trial.
Tajmiri et al., 2016 [14]	Nigella sativa	40 patients with HT	Randomized placebo-controlled trial; powdered N. sativa seeds 2 g/day; 8 wk	IL-23, TGF-beta, TSH, total T3/total T4, body weight	IL-23 and TSH decreased and total T3 increased; reported TPOAb/body-weight changes.	H1/H2; linked to [3,13], not an independent trial.
Farhangi et al., 2016 [3]	Nigella sativa	40 patients with HT	Randomized placebo-controlled trial; powdered N. sativa seeds 2 g/day; 8 wk	TSH, total T3, TPOAb, VEGF, nesfatin-1, BMI	TSH and TPOAb decreased, total T3 increased, with lower VEGF and anthropometric measures.	H1/H2; principal report of the same trial cluster as [13,14].
**(B)**						
**Study (Ref.)**	**Product/Intervention**	**Population/Model**	**Design/Exposure**	**Key Endpoints**	**Main Finding**	**Axis/Interpretive Role**
Kayumov et al., 2026 [16]	Quercetin, taxifolin, luteolin	Rat AIT model	Quercetin 25, taxifolin 20, luteolin 10 mg/kg/day, intragastric; 14 d	total T3/total T4/fT3, GPX1, minerals, microbiota	Luteolin produced the strongest restoration of thyroid-hormone/redox measures and microbiota profile.	H1/H2; promising but preclinical.
Wu et al., 2025 [17]	Citrus polymethoxyflavones (tangeretin/nobiletin)	Thyroid-cell models + mouse AIT	Tangeretin 4/20/100 mg/kg/day by oral gavage; 8 wk	TPOAb, IL-1beta/IL-6/TNF-alpha, IL-17 pathway, histology	Reduced TPOAb, inflammatory signaling and thyroid injury/infiltration.	H1; multi-level preclinical autoimmune evidence.
Kayumov et al., 2025 [18]	Quercetin, taxifolin, luteolin	30 male Wistar rats with AIT	Quercetin 25, taxifolin 20, luteolin 10 mg/kg/day; 14 d	TPOAb, TSH, thyroid histology, metabolic measures	All three improved AIT measures; luteolin showed the strongest overall effect.	H1/H2; direct AIT model.
Chen et al., 2024 [19]	Prunella vulgaris components: quercetin, luteolin, kaempferol, morin, beta-sitosterol	Primary human thyrocytes + Nthy-ori-3-1	In vitro; quercetin/luteolin/kaempferol/morin 10 or 40 μM; beta-sitosterol 50 or 150 μM; 24 h for cellular functional assays	TNF-alpha, IL-6, IFN-beta, apoptosis, NF-kB/IRF3, HSP90AA1	Key components reproduced anti-inflammatory/anti-apoptotic effects; HSP90AA1 identified as a target.	H1; high mechanistic relevance due to human thyrocytes.
Sun et al., 2021 [20]	Morin	Rat EAT model	Morin 50/100/200 mg/kg/day; 6 wk	TPOAb, TgAb, total T3/total T4, cytokines, oxidative stress, NLRP3/caspase-1	Improved EAT parameters and suppressed NLRP3/caspase-1 signaling.	H1/H2; inflammasome mechanism.
Guo et al., 2021 [21]	Prunella vulgaris/rosmarinic acid	Rat EAT + thyroid follicular cells	In vivo + in vitro; *P. vulgaris* extract 2 mL/kg/day in rats; 12 wk	TgAb, histology, cytokines, HMGB1/TLR9/MyD88, Th subsets	Reduced inflammation/TgAb and inhibited HMGB1/TLR9/MyD88 signaling.	H1; direct mechanistic EAT immunomodulatory model.
Qiu et al., 2020 [22]	Prunella vulgaris/rosmarinic acid	Lewis rats with EAT	*P. vulgaris* extract 1/2/4 mL/kg/day by intragastric administration; 12 wk	TgAb, histology, Treg, IL-10, TGF-beta, IDO1, Trp/Kyn	Reduced thyroiditis and TgAb; increased IDO1/Treg-related immunoregulation.	H1; IDO1-Treg mechanism.
Li, 2018 [23]	EGCG	Rat AIT model	EGCG 0.5 mg/kg i.p., three injections at 1 h intervals; AIT induction over 6 wk	Histology, IL-1beta, IFN-gamma, TNF-alpha, NF-kB, apoptosis/TRAIL	Reduced inflammatory and apoptotic signaling in AIT.	H1; anti-inflammatory/anti-apoptotic evidence.
Xia et al., 2016 [5]	Luteolin	Mouse EAT + RAW264.7 cells	Luteolin 10 mg/kg/day i.p. for 7 d; in vivo + in vitro	Histology, STAT3/STAT1, COX-2, TNF-alpha	Reduced lymphocytic infiltration/follicular injury and suppressed STAT3-related inflammation.	H1; coherent mechanistic support.
Hoshikawa et al., 2013 [24]	Green tea polyphenols	Iodide-induced AIT in NOD mice	0.2% green tea polyphenols with 0.05% iodide water; 8 wk	Thyroiditis, IL-10, serum total T4	IL-10 increased, but thyroiditis was not prevented and total T4 was unchanged.	H1/H2; important negative result.
Zhao et al., 2025 [25]	Buzhong Yiqi decoction	NOD.H-2h4 mouse AIT + Nthy-ori-3-1	Multicomponent botanical; in vivo + in vitro; dose and treatment duration not reported in the indexed abstract	Histology, TgAb/TPOAb, cytokines, oxidative stress, apoptosis, SIRT1/Nrf2/NF-kB	Improved histology and autoimmune/inflammatory/redox measures; reduced apoptosis.	H1; strong preclinical autoimmune/inflammatory signal for a complex mixture.

Abbreviations: AIT, autoimmune thyroiditis; BMI, body mass index; EAT, experimental autoimmune thyroiditis; EGCG, epigallocatechin-3-gallate; fT3, free triiodothyronine; fT4, free thyroxine; GPX, glutathione peroxidase; GPX1, glutathione peroxidase 1; H1, immune/redox axis; H2, direct thyroid-physiology axis; HbA1c, glycated hemoglobin; hs-CRP, high-sensitivity C-reactive protein; HSP90AA1, heat shock protein 90 alpha family class A member 1; HT, Hashimoto’s thyroiditis; ICAM-1, intercellular adhesion molecule 1; IDO1, indoleamine 2,3-dioxygenase 1; IFN, interferon; IL, interleukin; MDA, malondialdehyde; NF-kB, nuclear factor kappa B; NIS, sodium-iodide symporter; Nrf2, nuclear factor erythroid 2-related factor 2; RCT, randomized controlled trial; SIRT1, sirtuin 1; SOD, superoxide dismutase; TAC, total antioxidant capacity; TGF-beta, transforming growth factor beta; TgAb, thyroglobulin antibody; TNF-alpha, tumor necrosis factor alpha; TPO, thyroid peroxidase; TPOAb, thyroid peroxidase antibody; TRAIL, TNF-related apoptosis-inducing ligand; Treg, regulatory T cell; Trp/Kyn, tryptophan/kynurenine; TSH, thyroid-stimulating hormone; VCAM-1, vascular cell adhesion molecule 1; VEGF, vascular endothelial growth factor. Multiple reports from the same clinical trial are shown separately because they report different endpoint domains, but they are treated as one underlying trial in the interpretation.

**Table 3:** Methodological characteristics of the three independent clinical trials in Hashimoto’s thyroiditis.

Characteristic	Curcumin [4,11]	Nigella Sativa [3,13,14]	Genistein [15]
Design	Double-blind randomized placebo-controlled trial	Randomized placebo-controlled trial; linked reports from one cohort	Double-blind randomized placebo-controlled trial
Participants	62 randomized; 57 analyzed	47 randomized; 40 completed/analyzed	278 randomized; 218 included in reported analysis
Duration	12 weeks	8 weeks	1 month
Control/context	Placebo + same anti-inflammatory diet	Placebo	Placebo
Background thyroid treatment	Randomization stratified by LT4 use; thyroid medication common in both groups	Stable LT4 before and during trial	Background thyroid treatment/stability not sufficiently characterized in the available report
Intervention dose	Curcumin 1320 mg/day (3 × 440 mg curcuminoid capsules) + anti-inflammatory diet	Powdered Nigella sativa seeds 2 g/day (2 × 1 g capsules)	Genistein 600 mg/day (purified soy extract)
Baseline comparability/thyroid status	No significant baseline differences in reported characteristics; thyroid medication use 71.4% vs. 72.4%; randomization stratified by LT4 use and sex	No significant baseline differences in general characteristics; all participants received LT4 at a stable dose from ≥6 wk before enrollment through the trial	Female HT population; detailed baseline comparability and background thyroid treatment/stability were incompletely characterized in the available report
Adherence assessment	Weekly follow-up, capsule counts and dietary recalls; >10% deviation from prescribed diet or supplement led to exclusion from final analysis	Weekly telephone follow-up to encourage regular supplement use; no objective adherence measure reported	Not sufficiently reported in the available article
Adverse events/tolerability	No dedicated adverse-event analysis was clearly reported in the trial publications	Three intervention participants were excluded because of itching and nausea; no other side effects were reported	Described as well tolerated; detailed adverse-event data were not reported
Main endpoints	TPOAb, TSH, total T3/total T4; hs-CRP, IL-6, NF-kB	TSH, total T3/total T4, TPOAb; inflammatory, oxidative, vascular and metabolic markers across linked reports	total T3, total T4, fT4, TSH, TPOAb, TgAb, IFN-gamma, IL-2
Attrition/analysis	5/62 did not complete; non-adherent participants were excluded from final analysis	7/47 did not complete; completer analysis	60/278 (21.6%) post-randomization attrition
Clinical interpretation	Immunological/inflammatory signal; no demonstrated disease modification or patient-important benefit	Multiple surrogate signals from one small, short trial; not independent replication	Immunological and biochemical thyroid-function signals; no demonstrated disease modification
Principal limitations	Small sample; short duration; both groups received diet intervention; dietary adherence partly self-reported; per-protocol exclusion of non-adherent participants; surrogate outcomes; adverse-event reporting limited	Small sample; 8 weeks; completer analysis; multiple outcomes/publications from the same cohort; three intervention withdrawals due to itching/nausea; surrogate outcomes	Substantial attrition; female-only population; background thyroid therapy/baseline status incompletely characterized; detailed adverse-event reporting lacking; surrogate outcomes; no independent replication

**Table 4:** Compound-specific integration of H1 and H2 evidence.

Compound/Product	H1 Evidence	H2 Evidence	Human HT Evidence	Cross-Axis Interpretation/Major Uncertainty
Curcumin	Inflammatory markers and TPOAb signal	Thyroid-function endpoints assessed; no consistent adjusted TSH/total T3 superiority	One 57-patient analyzed RCT (two reports)	Preliminary immunological/biochemical signal; direct H2 mechanism and patient-important benefit insufficient
Nigella sativa	Oxidative, inflammatory and T-cell effects	TSH/total T3 changes reported	One 40-patient completer trial cluster + ex vivo study	Multiple linked reports are not independent replication; higher ex vivo exposure increased cell death
Genistein	TPOAb/TgAb and Th1-related cytokine changes	Biochemical thyroid-function changes in HT; experimental TPO inhibition	One trial; 218 included in reported analysis	Clear cross-model discordance; substantial attrition and no independent replication
Luteolin	Anti-inflammatory effects in AIT	Thyroid-hormone/functional measures in animal models	None	Preclinical concordance does not establish clinical efficacy
Quercetin	Anti-inflammatory/thyrocyte evidence in experimental systems	Experimental inhibition of thyroid-specific genes/function	None in HT	Potential cross-axis discordance; human exposure relevance uncertain
Resveratrol	HT-specific H1 evidence insufficient	Experimental NIS suppression/antithyroid activity	None in HT	Mechanistic signal does not establish clinical thyroid dysfunction
Prunella vulgaris	Multiple immunoregulatory/anti-inflammatory mechanisms	Direct standardized H2 evidence limited	None	Promising preclinical H1 cluster; complex-preparation translation uncertain
Rutin	Redox effects	Increased NIS expression/iodide uptake experimentally	None in HT	Enhanced iodide handling is not evidence of therapeutic benefit in HT
Apigenin	HT-specific H1 evidence insufficient	Enhanced TSH-stimulated radioiodide accumulation under defined conditions	None in HT	Mechanistic H2 finding; clinical relevance and exposure relevance uncertain

## Data Availability

No new experimental data were generated. The database exports, screening register, and evidence-classification files underlying this review are available from the corresponding author on reasonable request.

## Supplementary Materials

The following supporting information can be downloaded at: https://www.mdpi.com/article/10.3390/biomedicines14102273/s1, Table S1: Database-specific H1 and H2 search strategies and archived yields; Table S2: Study-level evidence register documenting evidence type, population/model, H1/H2 relevance, final evidence role, linked study-cluster relationships, and original/corrected evidence-type and evidence-level labels for records revised during the final audit.

## References

[1] Hu X., Chen Y., Shen Y., Tian R., Sheng Y., Que H. (2022). Global prevalence and epidemiological trends of Hashimoto’s thyroiditis in adults: A systematic review and meta-analysis. Front. Public Health.

[2] Chaker L., Papaleontiou M. (2025). Hypothyroidism: A review. JAMA.

[3] Farhangi M.A., Dehghan P., Tajmiri S., Abbasi M.M. (2016). The effects of Nigella sativa on thyroid function, serum vascular endothelial growth factor (VEGF)-1, Nesfatin-1 and anthropometric features in patients with Hashimoto’s thyroiditis: A randomized controlled trial. BMC Complement. Altern. Med..

[4] Bourbour F., Mahdavi B., Naghshi N., Yari Z., Moghimnejad Hosseini S., Kalbasi S., Sohrab G. (2026). The combined effects of an anti-inflammatory diet and curcumin supplementation on thyroid function and lipid profile in patients with Hashimoto’s thyroiditis: A double blind randomised clinical trial. Endocrinol. Diabetes Metab..

[5] Xia N., Chen G., Liu M., Ye X., Pan Y., Ge J., Mao Y., Wang H., Wang J., Xie S. (2016). Anti-inflammatory effects of luteolin on experimental autoimmune thyroiditis in mice. Exp. Ther. Med..

[6] Giuliani C., Iezzi M., Ciolli L., Hysi A., Bucci I., Di Santo S., Rossi C., Zucchelli M., Napolitano G. (2017). Resveratrol has anti-thyroid effects both in vitro and in vivo. Food Chem. Toxicol..

[7] Giuliani C., Di Dalmazi G., Bucci I., Napolitano G. (2024). Quercetin and thyroid. Antioxidants.

[8] Baethge C., Goldbeck-Wood S., Mertens S. (2019). SANRA-a scale for the quality assessment of narrative review articles. Res. Integr. Peer Rev..

[9] Kubiak K., Szmidt M.K., Kaluza J., Zylka A., Sicinska E. (2023). Do dietary supplements affect inflammation, oxidative stress, and antioxidant status in adults with hypothyroidism or Hashimoto’s disease? A systematic review of controlled trials. Antioxidants.

[10] Osowiecka K., Myszkowska-Ryciak J. (2023). The influence of nutritional intervention in the treatment of Hashimoto’s thyroiditis-a systematic review. Nutrients.

[11] Bourbour F., Mahdavi B., Naghshi N., Yari Z., Moghimnejad Hosseini S., Kalbasi S., Sohrab G. (2026). The additive effects of curcumin supplementation in addition to an anti-inflammatory diet on inflammatory indices in patients with Hashimoto’s thyroiditis (HT): A double blind randomized controlled clinical trial. Food Sci. Nutr..

[12] Ciesielska-Figlon K., Wojciechowicz K., Daca A., Kokotkiewicz A., Łuczkiewicz M., Witkowski J.M., Lisowska K.A. (2023). The impact of Nigella sativa essential oil on T cells in women with Hashimoto’s thyroiditis. Antioxidants.

[13] Abbasalizad Farhangi M., Tajmiri S. (2020). The effects of powdered black cumin seeds on markers of oxidative stress, intracellular adhesion molecule (ICAM)-1 and vascular cell adhesion molecule (VCAM)-1 in patients with Hashimoto’s thyroiditis. Clin. Nutr. ESPEN.

[14] Tajmiri S., Abbasalizad Farhangi M., Dehghan P. (2016). Nigella sativa treatment and serum concentrations of thyroid hormones, transforming growth factor beta (TGF-beta) and interleukin 23 (IL-23) in patients with Hashimoto’s thyroiditis. Eur. J. Integr. Med..

[15] Zhang K., Wang Y., Ma W., Hu Z., Zhao P. (2017). Genistein improves thyroid function in Hashimoto’s thyroiditis patients through regulating Th1 cytokines. Immunobiology.

[16] Kayumov K., Kuchkarova L., Romanova D., Szczuko M., Durá-Travé T., Ergashev N., Karimova I., Berdiyorova S., Pulatova V., Abdusamatov S. (2026). The restorative role of flavonoids in immunological, antioxidant, and microbiota mechanisms of autoimmune thyroiditis. Trends Sci..

[17] Wu M., Yang H., Wu Y., Wang D., Zheng Y., Sun C. (2025). Alleviating effects of citrus polymethoxyflavones on autoimmune thyroiditis via IL-17 signaling pathway. Food Front..

[18] Kayumov K., Kuchkarova L., Durá-Travé T., Petrenko S., Ergashev N., Abdurakhmonov J., Achilov R., Khan S., Pulatova V., Ernazarov M. (2025). Immunomodulatory and histoprotective effects of luteolin and related flavonoids in autoimmune thyroiditis: Evidence from a rat model. Trends Sci..

[19] Chen Y., Jiang B., Qu C., Jiang C., Zhang C., Wang Y., Chen F., Sun X., Su L., Luo Y. (2024). Bioactive components in Prunella vulgaris for treating Hashimoto’s disease via regulation of innate immune response in human thyrocytes. Heliyon.

[20] Sun Y.J., Zhang Y.F., Xu H.M., Ma Y.T., Li C., Nie C.H., Zhao M. (2021). Morin improves experimental autoimmune thyroiditis in rats via NLRP3/caspase-1 pathway. Sichuan Da Xue Xue Bao Yi Xue Ban.

[21] Guo Q., Qu H., Zhang H., Zhong X. (2021). Prunella vulgaris L. attenuates experimental autoimmune thyroiditis by inhibiting HMGB1/TLR9 signaling. Drug Des. Dev. Ther..

[22] Qiu H., Zhang J., Guo Q., Zhang Y., Zhong X. (2020). Prunella vulgaris L. attenuates experimental autoimmune thyroiditis by inducing indoleamine 2,3-dioxygenase 1 expression and regulatory T cell expansion. Biomed. Pharmacother..

[23] Li J. (2018). Neuroprotective effect of (-)-epigallocatechin-3-gallate on autoimmune thyroiditis in a rat model by an anti-inflammation effect, anti-apoptosis and inhibition of TRAIL signaling pathway. Exp. Ther. Med..

[24] Hoshikawa S., Nakagawa Y., Ozaki H., Takahashi Y., Ito S., Yoshida K., Mori K. (2013). Effects of green tea polyphenols on iodide-induced autoimmune thyroiditis in nonobese diabetic mice. Immunol. Investig..

[25] Zhao Z., Li J., Song N., Gao H., Liu D., Jin Z., Chen Y., Guo X., Liu Z., Yang X. (2025). Buzhong Yiqi decoction improves inflammation and oxidative damage in autoimmune thyroiditis by inhibiting apoptosis via the SIRT1-mediated Nrf2/NF-kB axis. J. Ethnopharmacol..

[26] Kayumov K., Kuchkarova L., Romanova D., Szczuko M., Berdiyorova S., Ergashev N., Karimova I., Yusupova U., Djabbarova G., Mirzakulov S. (2026). Effects of plant flavonoids and selenium on pancreatic hormones and glucose homeostasis in autoimmune thyroiditis. Exp. Anim..

[27] Tekieh M.F., Esfahanian F., Emadi F., Gholami M., Emaratkar E. (2019). Effect of Nigella sativa on thyroid function in patients with hypothyroidism treated with levothyroxine: A triple-blind randomized controlled trial. Med. Sci..

[28] Divi R.L., Chang H.C., Doerge D.R. (1997). Anti-thyroid isoflavones from soybean: Isolation, characterization, and mechanisms of action. Biochem. Pharmacol..

[29] Chang H.C., Doerge D.R. (2000). Dietary genistein inactivates rat thyroid peroxidase in vivo without an apparent hypothyroid effect. Toxicol. Appl. Pharmacol..

[30] Bitto A., Polito F., Atteritano M., Altavilla D., Mazzaferro S., Marini H., Adamo E.B., D’Anna R., Granese R., Corrado F. (2010). Genistein aglycone does not affect thyroid function: Results from a three-year, randomized, double-blind, placebo-controlled trial. J. Clin. Endocrinol. Metab..

[31] Hampl R., Ostatnikova D., Celec P., Putz Z., Lapcik O., Matucha P. (2008). Short-term effect of soy consumption on thyroid hormone levels and correlation with phytoestrogen level in healthy subjects. Endocr. Regul..

[32] Giuliani C., Bucci I., Di Santo S., Rossi C., Grassadonia A., Piantelli M., Monaco F., Napolitano G. (2014). The flavonoid quercetin inhibits thyroid-restricted genes expression and thyroid function. Food Chem. Toxicol..

[33] Giuliani C., Bucci I., Di Santo S., Rossi C., Grassadonia A., Mariotti M., Piantelli M., Monaco F., Napolitano G. (2014). Resveratrol inhibits sodium/iodide symporter gene expression and function in rat thyroid cells. PLoS ONE.

[34] Lima Goncalves C.F., de Souza dos Santos M.C., Ginabreda M.G., Soares Fortunato R., Pires de Carvalho D., Freitas Ferreira A.C. (2013). Flavonoid rutin increases thyroid iodide uptake in rats. PLoS ONE.

[35] Lima Goncalves C.F., de Freitas M.L., Fortunato R.S., Miranda-Alves L., Carvalho D.P., Ferreira A.C.F. (2018). Rutin scavenges reactive oxygen species, inactivates 5′-adenosine monophosphate-activated protein kinase, and increases sodium-iodide symporter expression in thyroid PCCL3 cells. Thyroid.

[36] Lakshmanan A., Doseff A.I., Ringel M.D., Saji M., Rousset B., Zhang X., Jhiang S.M. (2014). Apigenin in combination with Akt inhibition significantly enhances thyrotropin-stimulated radioiodide accumulation in thyroid cells. Thyroid.

[37] Psarias G., Ziros P.G., Mageiropoulou A., Chartoumpekis D.V., Habeos G.I., Alomair B.M., Duntas L., Trougakos I.P., Sykiotis G.P. (2026). Limited Nrf2 activation and heterogeneous thyroidal effects in a 424-compound multi-assay screen call for rigorous testing of purported antioxidant and health-promoting supplements. Redox Biol..

