# Supplementary material for: Natural Products in Hashimoto’s Thyroiditis: Therapeutic and Thyroid-Disrupting Effects—A Critical Narrative Review
Source: Biomedicines. 2026 Oct 8;14(10):2273. doi: 10.3390/biomedicines14102273 (PMC13701731; doi:10.3390/biomedicines14102273)
Supplement: Supplementary file 1 [file biomedicines-14-02273-s001.zip › biomedicines-4607038-supplementary.pdf]

Supplementary Materials

Natural Products in Hashimoto’s Thyroiditis: Therapeutic and Thyroid-Disrupting Effects—A Critical Narrative Review

Dorota Szydlarska1,2, Marta Zuzanna Ciechomska1, Zuzanna Banaszkiewicz2,*, Marek Brytan3, Justyna Chałdaś-Majdańska5, Melania Brzozowska4, Andrzej Śliwczyński5

Supplementary Table S1. Database-specific H1 and H2 search strategies and archived yields (search date: 24 August 2026).

Database	Axis	Search string	Archived yield
PubMed/MEDLINE	H1	(("Hashimoto Thyroiditis"[Mesh] OR "Hashimoto thyroiditis"[tiab] OR "Hashimoto's thyroiditis"[tiab] OR "autoimmune thyroiditis"[tiab] OR "autoimmune hypothyroidism"[tiab]) AND ("natural product*"[tiab] OR phytochemical*[tiab] OR polyphenol*[tiab] OR flavonoid*[tiab] OR alkaloid*[tiab] OR curcumin[tiab] OR "Nigella sativa"[tiab] OR genistein[tiab] OR quercetin[tiab] OR luteolin[tiab] OR resveratrol[tiab] OR "Prunella vulgaris"[tiab] OR catechin*[tiab] OR EGCG[tiab]) AND ("oxidative stress"[tiab] OR antioxidant*[tiab] OR inflammation[tiab] OR inflammatory[tiab] OR immunomodulat*[tiab] OR cytokine*[tiab] OR NF-kB[tiab] OR Nrf2[tiab] OR Th17[tiab] OR Treg[tiab] OR Tfh[tiab] OR Tfr[tiab] OR "TNF-alpha"[tiab] OR "IL-17"[tiab] OR apoptosis[tiab]))	23
PubMed/MEDLINE	H2	((thyroid[tiab] OR thyrocyte*[tiab] OR "thyroid follicular cell*"[tiab]) AND ("natural product*"[tiab] OR phytochemical*[tiab] OR polyphenol*[tiab] OR flavonoid*[tiab] OR alkaloid*[tiab] OR curcumin[tiab] OR "Nigella sativa"[tiab] OR genistein[tiab] OR quercetin[tiab] OR luteolin[tiab] OR resveratrol[tiab] OR "Prunella vulgaris"[tiab] OR catechin*[tiab] OR EGCG[tiab]) AND ("thyroid peroxidase"[tiab] OR TPO[tiab] OR "sodium iodide symporter"[tiab] OR NIS[tiab] OR "iodide uptake"[tiab] OR thyroglobulin[tiab] OR "TSH receptor"[tiab] OR TSHR[tiab] OR "thyroid hormone synthesis"[tiab] OR "thyroid hormone*"[tiab] OR "thyroid-specific gene*"[tiab] OR PAX8[tiab] OR NKX2-1[tiab] OR FOXE1[tiab]))	122
Scopus	H1	TITLE-ABS-KEY(("Hashimoto thyroiditis" OR "Hashimoto's thyroiditis" OR "autoimmune thyroiditis" OR "autoimmune hypothyroidism")) AND TITLE-ABS-KEY(("natural product*" OR phytochemical* OR polyphenol* OR flavonoid* OR alkaloid* OR curcumin OR "Nigella sativa" OR genistein OR quercetin OR luteolin OR resveratrol OR "Prunella vulgaris" OR catechin* OR EGCG)) AND TITLE-ABS-KEY(("oxidative stress" OR antioxidant* OR inflammation OR inflammatory OR immunomodulat* OR cytokine* OR NF-kB OR Nrf2 OR Th17 OR Treg OR Tfh OR Tfr OR "TNF-alpha" OR "IL-17" OR apoptosis))	74
Scopus	H2	TITLE-ABS-KEY((thyroid OR thyrocyte* OR "thyroid follicular cell*")) AND TITLE-ABS-KEY(("natural product*" OR phytochemical* OR polyphenol* OR flavonoid* OR alkaloid* OR curcumin OR "Nigella sativa" OR genistein OR quercetin OR luteolin OR resveratrol OR "Prunella vulgaris" OR catechin* OR EGCG)) AND TITLE-ABS-KEY(("thyroid peroxidase" OR TPO OR "sodium iodide symporter" OR NIS OR "iodide uptake" OR thyroglobulin OR "TSH receptor" OR TSHR OR "thyroid hormone synthesis" OR "thyroid hormone*" OR "thyroid-specific gene*" OR PAX8 OR NKX2-1 OR FOXE1))	263
Web of Science	H1	TS=(("Hashimoto thyroiditis" OR "Hashimoto's thyroiditis" OR "autoimmune thyroiditis" OR "autoimmune hypothyroidism")) AND TS=(("natural product*" OR phytochemical* OR polyphenol* OR flavonoid* OR alkaloid* OR curcumin OR "Nigella sativa" OR genistein OR quercetin OR luteolin OR resveratrol OR "Prunella vulgaris" OR catechin* OR EGCG)) AND TS=(("oxidative stress" OR antioxidant* OR inflammation OR inflammatory OR immunomodulat* OR cytokine* OR NF-kB OR Nrf2 OR Th17 OR Treg OR Tfh OR Tfr OR "TNF-alpha" OR "IL-17" OR apoptosis))	34
Web of Science	H2	TS=((thyroid OR thyrocyte* OR "thyroid follicular cell*")) AND TS=(("natural product*" OR phytochemical* OR polyphenol* OR flavonoid* OR alkaloid* OR curcumin OR "Nigella sativa" OR genistein OR quercetin OR luteolin OR resveratrol OR "Prunella vulgaris" OR catechin* OR EGCG)) AND TS=(("thyroid peroxidase" OR TPO OR "sodium iodide symporter" OR NIS OR "iodide uptake" OR thyroglobulin OR "TSH receptor" OR TSHR OR "thyroid hormone synthesis" OR "thyroid hormone*" OR "thyroid-specific gene*" OR PAX8 OR NKX2-1 OR FOXE1))	171

Search-strategy note. The strings shown in Supplementary Table S1 are the database-specific strategies used in the project. Archived yields correspond to searches completed on 24 August 2026, and the associated database exports were retained in the project archive.

Supplementary Table S2. Study-level evidence register and final evidence-role classification.

The final retained evidence set comprised 79 publications: 18 core HT/AIT publications, 56 supportive thyroid-physiology publications, and 5 contextual publications. Corrected evidence-type and evidence-level labels were used in the final synthesis. For records revised during the final audit, the original labels are shown in the last two columns for traceability. Records retained as uncertain/background material during screening are not included in Supplementary Table S2 because they were outside the 79-publication final evidence set and were not used as primary evidence in the principal synthesis; they remain in the screening archive for traceability and contextual citation chasing. Terminology note: TPOAb and TgAb denote circulating autoantibodies and are classified here as autoimmune/immunological outcomes (H1), not as direct H2 effects on TPO or thyroglobulin. H2:TPO is reserved for direct TPO enzyme activity/expression and H2:thyroglobulin for direct thyroglobulin expression or protein.

Panel A. Core HT/AIT evidence

Study / DOI	Final evidence type	Final level	Population / model	H1/H2 relevance	Final evidence role	Linked study cluster	Original evidence type (audit only)	Original level (audit only)
The Additive Effects of Curcumin Supplementation in Addition to an Anti-Inflammatory Diet on Inflammatory Indices in Patients With Hashimoto's Thyroiditis (HT): A Double Blind Randomized Controlled Clinical Trial. (2026)<br>DOI: 10.1002/fsn3.71572	Human RCT / controlled trial	A	57 patients with HT	H1: inflammation/immunomodulation	Core HT/AIT evidence	Curcumin RCT NCT05975866 (two reports)	—	—
The Combined Effects of an Anti-Inflammatory Diet and Curcumin Supplementation on Thyroid Function and Lipid Profile in Patients With Hashimoto's Thyroiditis: A Double Blind Randomised Clinical Trial. (2026)<br>DOI: 10.1002/edm2.70138	Human RCT / controlled trial	A	Patients with HT	H1: thyroid autoimmunity (TPOAb); H2: thyroid hormone synthesis/function	Core HT/AIT evidence	Curcumin RCT NCT05975866 (two reports)	—	—
The Restorative Role of Flavonoids in Immunological, Antioxidant, and Microbiota Mechanisms of Autoimmune Thyroiditis (2026)<br>DOI: 10.48048/tis.2026.12516	Animal model	C	Rat AIT model	H1: oxidative stress/antioxidant; H1: inflammation/immunomodulation; H2: thyroid hormone synthesis/function	Core HT/AIT evidence	—	—	—
Alleviating Effects of Citrus Polymethoxyflavones on Autoimmune Thyroiditis via IL-17 Signaling Pathway (2025)<br>DOI: 10.1002/fft2.70053	In vitro - human thyrocytes	B	Thyroid-cell models + mouse AIT	H1: inflammation/immunomodulation; H1: thyroid autoimmunity (TPOAb)	Core HT/AIT evidence	—	—	—
Buzhong Yiqi decoction improves inflammation and oxidative damage in autoimmune thyroiditis by inhibiting apoptosis via the SIRT1-Mediated Nrf2/NF-κB axis (2025)<br>DOI: 10.1016/j.jep.2025.119967	Animal + in vitro	C	NOD.H-2h4 mouse AIT + Nthy-ori-3-1	H1: oxidative stress/antioxidant; H1: inflammation/immunomodulation; H1: thyrocyte apoptosis/survival; H1: thyroid autoimmunity (TPOAb/TgAb)	Core HT/AIT evidence	—	Human thyrocytes in vitro	B
Immunomodulatory and Histoprotective Effects of Luteolin and Related Flavonoids in Autoimmune Thyroiditis: Evidence from A Rat Model (2025)<br>DOI: 10.48048/tis.2026.10981	Animal model	C	30 male Wistar rats with AIT	H1: oxidative stress/antioxidant; H1: inflammation/immunomodulation; H1: thyroid autoimmunity (TPOAb); H2: thyroid hormone synthesis/function	Core HT/AIT evidence	—	—	—
Bioactive components in prunella vulgaris for treating Hashimoto's disease via regulation of innate immune response in human thyrocytes. (2024)<br>DOI: 10.1016/j.heliyon.2024.e36103	In vitro - human thyrocytes	B	Primary human thyrocytes + Nthy-ori-3-1	H1: oxidative stress/antioxidant; H1: inflammation/immunomodulation; H1: thyrocyte apoptosis/survival	Core HT/AIT evidence	—	—	—
The Impact of Nigella sativa Essential Oil on T Cells in Women with Hashimoto's Thyroiditis. (2023)<br>DOI: 10.3390/antiox12061246	Ex vivo - HT patient lymphocytes	B	T lymphocytes from women with HT and healthy controls	H1: inflammation/immunomodulation; H1: immune-cell viability/apoptosis	Core HT/AIT evidence	—	Experimental / other	D
Morin improves experimental autoimmune thyroiditis in rats via nlrp3/caspase-1 pathway (2021)<br>DOI: 10.12182/20210160507	Animal model	C	Rat EAT model	H1: oxidative stress/antioxidant; H1: inflammation/immunomodulation; H1: thyrocyte apoptosis/survival;	Core HT/AIT evidence	—	—	—

Study / DOI	Final evidence type	Final level	Population / model	H1/H2 relevance	Final evidence role	Linked study cluster	Original evidence type (audit only)	Original level (audit only)
				H1: thyroid autoimmunity (TPOAb/TgAb); H2: thyroid hormone synthesis/function				
Prunella vulgaris L. Attenuates Experimental Autoimmune Thyroiditis by Inhibiting HMGB1/TLR9 Signaling. (2021) DOI: 10.2147/dddt.s325814	Animal + in vitro	C	Rat EAT + thyroid follicular cells	H1: oxidative stress/antioxidant; H1: inflammation/immunomodulation; H1: thyroid autoimmunity (TgAb)	Core HT/AIT evidence	—	In vitro / cell model	C
Prunella vulgaris L. attenuates experimental autoimmune thyroiditis by inducing indoleamine 2,3-dioxygenase 1 expression and regulatory T cell expansion. (2020) DOI: 10.1016/j.biopha.2020.110288	Animal model	C	Lewis rats with EAT	H1: oxidative stress/antioxidant; H1: inflammation/immunomodulation; H1: thyroid autoimmunity (TgAb)	Core HT/AIT evidence	—	Experimental / other	D
The effects of powdered black cumin seeds on markers of oxidative stress, intracellular adhesion molecule (ICAM)-1 and vascular cell adhesion molecule (VCAM)-1 in patients with Hashimoto's thyroiditis. (2020) DOI: 10.1016/j.clnesp.2020.02.015	Human RCT / controlled trial	A	40 patients with HT	H1: oxidative stress/antioxidant	Core HT/AIT evidence	Nigella sativa 40-patient, 8-week trial cluster (IRCT2015021719082N4)	—	—
Neuroprotective effect of (-)-epigallocatechin-3-gallate on autoimmune thyroiditis in a rat model by an anti-inflammation effect, anti-apoptosis and inhibition of TRAIL signaling pathway. (2018) DOI: 10.3892/etm.2017.5511	Animal model	C	Rat AIT model	H1: oxidative stress/antioxidant; H1: inflammation/immunomodulation; H1: thyrocyte apoptosis/survival	Core HT/AIT evidence	—	—	—
Genistein improves thyroid function in Hashimoto's thyroiditis patients through regulating Th1 cytokines. (2017) DOI: 10.1016/j.imbio.2016.10.004	Human RCT	A	218 patients with HT	H1: inflammation/immunomodulation; H1: thyroid autoimmunity (TPOAb/TgAb); H2: thyroid hormone synthesis/function	Core HT/AIT evidence	—	Nonrandomized human/exposure	B
Anti-inflammatory effects of luteolin on experimental autoimmune thyroiditis in mice. (2016) DOI: 10.3892/etm.2016.3854	Animal model	C	Mouse model with autoimmune thyroiditis	H1: oxidative stress/antioxidant; H1: inflammation/immunomodulation; H2: thyroid hormone synthesis/function	Core HT/AIT evidence	—	—	—
Nigella Sativa treatment and serum concentrations of thyroid hormones, transforming growth factor β (TGF-β) and interleukin 23 (IL-23) in patients with Hashimoto's Thyroiditis (2016) DOI: 10.1016/j.eujim.2016.03.003	Human RCT	A	40 patients with HT	H1: inflammation/immunomodulation; H1: thyroid autoimmunity (TPOAb); H2: thyroid hormone synthesis/function	Core HT/AIT evidence	Nigella sativa 40-patient, 8-week trial cluster (IRCT2015021719082N4)	Nonrandomized human/exposure	B
The effects of Nigella sativa on thyroid function, serum Vascular Endothelial Growth Factor (VEGF) - 1, Nesfatin-1 and anthropometric features in patients with Hashimoto's thyroiditis: a randomized controlled trial. (2016) DOI: 10.1186/s12906-016-1432-2	Human RCT / controlled trial	A	Patients with HT	H1: inflammation/immunomodulation; H1: thyroid autoimmunity (TPOAb); H2: thyroid hormone synthesis/function	Core HT/AIT evidence	Nigella sativa 40-patient, 8-week trial cluster (IRCT2015021719082N4)	—	—
Effects of green tea polyphenols on iodide-induced autoimmune thyroiditis in nonobese diabetic mice. (2013) DOI: 10.3109/08820139.2012.753611	Animal model	C	Iodide-induced AIT in NOD mice	H1: inflammation/immunomodulation; H2: thyroid hormone synthesis/function	Core HT/AIT evidence	—	—	—

Panel B. Supportive thyroid-physiology evidence

Study / DOI	Final evidence type	Final level	Population / model	H1/H2 relevance	Final evidence role	Linked study cluster	Original evidence type (audit only)	Original level (audit only)
Limited Nrf2 activation and heterogeneous thyroidal effects in a 424-compound multi-assay screen call for rigorous testing of purported antioxidant and health-promoting supplements. (2026)<br>DOI: 10.1016/j.redox.2026.104222	In vitro / cell model	C	Thyroid-cell model	H1: oxidative stress/antioxidant; H1: inflammation/immunomodulation; H2: NIS/iodide uptake; H2: thyroglobulin	Supportive thyroid-physiology evidence	—	—	—
Withania somnifera Root Extract Ameliorates PTU-Induced Hypothyroidism by Regulating Hormone Levels and Gene Expression in Rats. (2026)<br>DOI: 10.1002/fsn3.71732	Animal model	C	Rat model with PTU-induced thyroid dysfunction	H1: oxidative stress/antioxidant; H1: inflammation/immunomodulation; H2: TPO; H2: thyroglobulin; H2: TSHR; H2: thyroid hormone synthesis/function	Supportive thyroid-physiology evidence	—	—	—
Animals in Levothyroxine Model of Thyroid Damage Are Positively Affected by the Consumption of Descurainia sophia L. Extract (Brassicaceae) (2025)<br>DOI: 10.22037/ijps.v21i1.46573	Animal model	C	Rat model with hyperthyroidism	H2: NIS/iodide uptake; H2: thyroid hormone synthesis/function	Supportive thyroid-physiology evidence	—	—	—
Antioxidant activity and thyroid peroxidase inhibition of Allii macrostemonis bulbus extracts (2025)<br>DOI: 10.1117/12.3095494	Experimental / other	D	Experimental/mechanistic model	H1: oxidative stress/antioxidant; H1: inflammation/immunomodulation; H2: TPO	Supportive thyroid-physiology evidence	—	—	—
Conocarpus erectus extracts restore thyroid architecture and downregulate pro-apoptotic markers in propylthiouracil-treated rats (2025)<br>DOI: 10.1016/j.phyplu.2025.100913	Animal model	C	Rat model with PTU-induced thyroid dysfunction	H1: oxidative stress/antioxidant; H1: inflammation/immunomodulation; H1: thyrocyte apoptosis/survival; H2: thyroid hormone synthesis/function	Supportive thyroid-physiology evidence	—	Human RCT	A
Ameliorative effect of ferulic acid on thyroid dysfunction against propyl-thiouracil induced hypothyroid rats (2024)<br>DOI: 10.1007/s12020-024-03818-z	Animal model	C	Rat model with PTU-induced thyroid dysfunction	H1: oxidative stress/antioxidant; H1: inflammation/immunomodulation; H2: TPO; H2: thyroglobulin; H2: thyroid hormone synthesis/function	Supportive thyroid-physiology evidence	—	—	—
Curcumin exerts protective effects on the thyroid gland in propylthiouracil-treated rats (2023)<br>DOI: —	Animal model	C	Rat model with PTU-induced thyroid dysfunction	H2: thyroid hormone synthesis/function	Supportive thyroid-physiology evidence	—	—	—
Peripubertal soy isoflavone consumption leads to subclinical hypothyroidism in male Wistar rats. (2023)<br>DOI: 10.1017/s2040174422000496	Animal model	C	Rat model with hypothyroidism	H2: NIS/iodide uptake; H2: thyroid hormone synthesis/function	Supportive thyroid-physiology evidence	—	—	—
Effects of soy-containing infant formulas on thyroid gland in rats (2022)<br>DOI: 10.23736/s2724-5276.21.06221-6	Animal model	C	Rat model with hyperthyroidism	H1: inflammation/immunomodulation; H2: thyroid hormone synthesis/function	Supportive thyroid-physiology evidence	—	Nonrandomized human/exposure	B
Investigating the impact of resveratrol on thyroid function, structure, and metabolic alterations in hyperthyroidism model: in vivo study (2022)<br>DOI: 10.47750/pnr.2022.13.04.025	Animal model	C	Rat model with hyperthyroidism	H1: oxidative stress/antioxidant; H1: inflammation/immunomodulation; H2: NIS/iodide uptake; H2: thyroid hormone synthesis/function	Supportive thyroid-physiology evidence	—	—	—
Possible mechanism of bamboo shoots (Bambusa balcooa) induced thyroid disruption - An in vitro study. (2021)<br>DOI: 10.1177/0960327120958037	In vitro / cell model	C	Thyroid-cell model	H1: oxidative stress/antioxidant; H2: thyroglobulin	Supportive thyroid-physiology evidence	—	—	—
Resveratrol Alleviates the Inhibitory Effect of Tunicamycin-Induced Endoplasmic Reticulum Stress on Expression of Genes Involved in Thyroid	In vitro / cell model	C	Thyroid-cell model	H2: TPO; H2: NIS/iodide uptake; H2: thyroglobulin; H2: TSHR; H2: thyroid hormone synthesis/function	Supportive thyroid-physiology evidence	—	—	—

Study / DOI	Final evidence type	Final level	Population / model	H1/H2 relevance	Final evidence role	Linked study cluster	Original evidence type (audit only)	Original level (audit only)
Hormone Synthesis in FRTL-5 Thyrocytes. (2021)<br>DOI: 10.3390/ijms22094373								
Some Dietary Phenolic Compounds Can Activate Thyroid Peroxidase and Inhibit Lipoxygenase-Preliminary Study in the Model Systems. (2021)<br>DOI: 10.3390/ijms22105108	Experimental / other	D	Experimental/mechanistic model	H1: oxidative stress/antioxidant; H2: TPO	Supportive thyroid-physiology evidence	—	—	—
Biologically-Guided Isolation of Natural Lead Antithyroid Drug from Medicago sativa L. Sprouts and Its Toxic Profile in Comparison with Propylthiouracil. (2020)<br>DOI: 10.38212/2224-6614.1242	In vitro / cell model	C	Cell/biochemical model	H1: oxidative stress/antioxidant; H2: TPO; H2: thyroid hormone synthesis/function	Supportive thyroid-physiology evidence	—	—	—
Effects of bamboo shoots (Bambusa balcooa) on thyroid hormone synthesizing regulatory elements at cellular and molecular levels in thyrocytes. (2020)<br>DOI: 10.1016/j.jep.2019.112463	In vitro / cell model	C	Thyroid-cell model	H1: oxidative stress/antioxidant; H1: inflammation/immunomodulation; H2: TPO	Supportive thyroid-physiology evidence	—	—	—
Broccoli sprout beverage is safe for thyroid hormonal and autoimmune status: Results of a 12-week randomized trial. (2019)<br>DOI: 10.1016/j.fct.2019.02.004	Human RCT / controlled trial	A	Human participants	H1: oxidative stress/antioxidant; H1: thyroid autoimmunity (TPOAb/TgAb); H2: thyroglobulin; H2: thyroid hormone synthesis/function	Supportive thyroid-physiology evidence	—	—	—
Effect of Nigella sativa on thyroid function in patients with hypothyroidism treated with levothyroxine: a triple-blind randomized controlled trial (2019)<br>DOI: —	Human RCT / controlled trial	A	Patients with hypothyroidism	H1: thyroid autoimmunity (TPOAb); H2: thyroid hormone synthesis/function	Supportive thyroid-physiology evidence	—	—	—
Goitrogenic/antithyroidal potential of moringa leaves (Moringa oleifera) and spinach (spinacia oleracea) of indian origin on thyroid status in male albino rats (2019)<br>DOI: 10.1590/s2175-97902019000218005	Animal model	C	Rat model with hypothyroidism	H2: thyroid hormone synthesis/function	Supportive thyroid-physiology evidence	—	—	—
Mechanism of Action and Interactions between Thyroid Peroxidase and Lipoxygenase Inhibitors Derived from Plant Sources. (2019)<br>DOI: 10.3390/biom9110663	In vitro / cell model	C	Cell/biochemical model	H1: oxidative stress/antioxidant; H2: TPO	Supportive thyroid-physiology evidence	—	—	—
The Flavonoid Quercetin Induces AP-1 Activation in FRTL-5 Thyroid Cells. (2019)<br>DOI: 10.3390/antiox8050112	In vitro / cell model	C	Thyroid-cell model	H2: thyroid hormone synthesis/function	Supportive thyroid-physiology evidence	—	—	—
Thyroid Peroxidase Activity is Inhibited by Phenolic Compounds-Impact of Interaction. (2019)<br>DOI: 10.3390/molecules24152766	In vitro / cell model	C	Cell/biochemical model	H1: oxidative stress/antioxidant; H2: TPO	Supportive thyroid-physiology evidence	—	—	—
Rutin Scavenges Reactive Oxygen Species, Inactivates 5'-Adenosine Monophosphate-Activated Protein Kinase, and Increases Sodium-Iodide Symporter Expression in Thyroid PCCL3 Cells. (2018)<br>DOI: 10.1089/thy.2016.0585	In vitro / cell model	C	Thyroid-cell model	H1: oxidative stress/antioxidant; H2: NIS/iodide uptake; H2: thyroid hormone synthesis/function	Supportive thyroid-physiology evidence	—	—	—
The phytoestrogen genistein prevents trabecular bone loss and affects thyroid follicular cells in a male rat model of osteoporosis. (2018)<br>DOI: 10.1111/joa.12828	Animal model	C	Rat model	H1: oxidative stress/antioxidant; H2: thyroglobulin; H2: thyroid hormone synthesis/function	Supportive thyroid-physiology evidence	—	—	—
Citrus flavanones mildly interfere with pituitary -thyroid axis in old-aged male rats (2017)<br>DOI: 10.1016/j.acthis.2017.02.005	Animal model	C	Rat model	H1: oxidative stress/antioxidant; H2: thyroglobulin; H2: thyroid hormone synthesis/function	Supportive thyroid-physiology evidence	—	—	—
Effect of curcumin in prolonging survival time of cryopreserved mice via promoting thyroid function	Animal model	C	Mouse model with PTU-induced thyroid dysfunction	H2: TPO; H2: NIS/iodide uptake; H2: thyroglobulin; H2: thyroid	Supportive thyroid-physiology evidence	—	—	—

Study / DOI	Final evidence type	Final level	Population / model	H1/H2 relevance	Final evidence role	Linked study cluster	Original evidence type (audit only)	Original level (audit only)
(2017)<br>DOI: 10.3969/j.issn.1001-1978.2017.09.020				hormone synthesis/function				
Resveratrol has anti-thyroid effects both in vitro and in vivo. (2017)<br>DOI: 10.1016/j.fct.2017.06.044	In vitro / cell model	C	Thyroid-cell model	H1: oxidative stress/antioxidant; H1: inflammation/immunomodulation; H2: TPO; H2: NIS/iodide uptake; H2: thyroglobulin; H2: TSHR	Supportive thyroid-physiology evidence	—	—	—
Studies on goitrogenic/ antithyroidal potentiality of thiocyanate, catechin and after concomitant exposure of thiocyanate-catechin (2016)<br>DOI: —	Experimental / other	D	Experimental/mechanistic model	H2: TPO; H2: thyroid hormone synthesis/function	Supportive thyroid-physiology evidence	—	—	—
Synergic actions of polyphenols and cyanogens of peanut seed coat (Arachis hypogaea) on cytological, biochemical and functional changes in thyroid. (2015)<br>DOI: —	Experimental / other	D	Experimental/mechanistic model	H2: TPO; H2: thyroid hormone synthesis/function	Supportive thyroid-physiology evidence	—	—	—
Antithyroid effects of naringin, hesperidin and rutin in L-T4 induced hyperthyroid rats: Possible mediation through 5′DI activity (2014)<br>DOI: 10.1016/j.pharep.2014.07.002	Animal model	C	Rat model with hyperthyroidism	H1: oxidative stress/antioxidant; H2: thyroid hormone synthesis/function	Supportive thyroid-physiology evidence	—	—	—
Apigenin in combination with Akt inhibition significantly enhances thyrotropin-stimulated radioiodide accumulation in thyroid cells. (2014)<br>DOI: 10.1089/thy.2013.0614	In vitro / cell model	C	Thyroid-cell model	H2: NIS/iodide uptake	Supportive thyroid-physiology evidence	—	—	—
Resveratrol inhibits sodium/iodide symporter gene expression and function in rat thyroid cells. (2014)<br>DOI: 10.1371/journal.pone.0107936	Animal model	C	Rat model	H1: oxidative stress/antioxidant; H1: inflammation/immunomodulation; H2: NIS/iodide uptake; H2: thyroid hormone synthesis/function	Supportive thyroid-physiology evidence	—	—	—
Soy isoflavones interfere with thyroid hormone homeostasis in orchidectomized middle-aged rats. (2014)<br>DOI: 10.1016/j.taap.2014.04.018	Animal model	C	Rat model	H2: TPO; H2: thyroglobulin; H2: thyroid hormone synthesis/function	Supportive thyroid-physiology evidence	—	—	—
The flavonoid quercetin inhibits thyroid-restricted genes expression and thyroid function. (2014)<br>DOI: 10.1016/j.fct.2014.01.016	Experimental / other	D	Experimental/mechanistic model	H1: oxidative stress/antioxidant; H1: inflammation/immunomodulation; H2: TPO; H2: NIS/iodide uptake; H2: thyroglobulin; H2: TSHR; H2: thyroid hormone synthesis/function	Supportive thyroid-physiology evidence	—	—	—
Catechin induced modulation in the activities of thyroid hormone synthesizing enzymes leading to hypothyroidism. (2013)<br>DOI: 10.1007/s11010-012-1503-8	In vitro / cell model	C	Cell/biochemical model	H1: oxidative stress/antioxidant; H1: inflammation/immunomodulation; H2: TPO; H2: thyroid hormone synthesis/function	Supportive thyroid-physiology evidence	—	—	—
Flavonoid rutin increases thyroid iodide uptake in rats. (2013)<br>DOI: 10.1371/journal.pone.0073908	Animal model	C	Rat model	H2: TPO; H2: NIS/iodide uptake; H2: TSHR; H2: thyroid hormone synthesis/function	Supportive thyroid-physiology evidence	—	—	—
Soy extracts suppressed iodine uptake and stimulated the production of autoimmunogen in rat thyrocytes. (2013)<br>DOI: 10.1177/1535370213489488	Animal model	C	Rat model with hypothyroidism	H1: inflammation/immunomodulation; H2: NIS/iodide uptake; H2: thyroglobulin	Supportive thyroid-physiology evidence	—	—	—
The presence of monoiodinated derivates of daidzein and genistein in human urine and its effect on thyroid gland function. (2012)<br>DOI: 10.1016/j.fct.2012.05.037	Experimental / other	D	Experimental/mechanistic model	H2: TPO	Supportive thyroid-physiology evidence	—	—	—

Study / DOI	Final evidence type	Final level	Population / model	H1/H2 relevance	Final evidence role	Linked study cluster	Original evidence type (audit only)	Original level (audit only)
Effect of different doses of un-fractionated green and black tea extracts on thyroid physiology. (2011)<br>DOI: 10.1177/0960327110382563	Experimental / other	D	Experimental/mechanistic model	H2: TPO; H2: thyroid hormone synthesis/function	Supportive thyroid-physiology evidence	—	—	—
Effects of dietary soy intake on maternal thyroid functions and serum anti-thyroperoxidase antibody level during early pregnancy. (2011)<br>DOI: 10.1089/jmf.2010.1078	Nonrandomized human / exposure study	B	Human participants / exposure study	H1: thyroid autoimmunity (TPOAb); H2: thyroid hormone synthesis/function	Supportive thyroid-physiology evidence	—	—	—
Genistein aglycone does not affect thyroid function: results from a three-year, randomized, double-blind, placebo-controlled trial. (2010)<br>DOI: 10.1210/jc.2009-2779	Human RCT / controlled trial	A	Patients with hypothyroidism	H1: thyroid autoimmunity (TPOAb/TgAb); H2: thyroid hormone synthesis/function	Supportive thyroid-physiology evidence	—	—	—
Goitrogenic/antithyroidal potential of green tea extract in relation to catechin in rats. (2010)<br>DOI: 10.1016/j.fct.2010.05.064	Animal model	C	Rat model	H2: TPO; H2: thyroid hormone synthesis/function	Supportive thyroid-physiology evidence	—	—	—
Resveratrol increases iodide trapping in the rat thyroid cell line FRTL-5. (2010)<br>DOI: 10.1089/thy.2009.0171	Animal model	C	Rat model	H1: oxidative stress/antioxidant; H1: inflammation/immunomodulation; H2: NIS/iodide uptake	Supportive thyroid-physiology evidence	—	—	—
Suppressive effects of genistein and daidzein on pituitary-thyroid axis in orchidectomized middle-aged rats. (2010)<br>DOI: 10.1258/ebm.2009.009279	Animal model	C	Rat model	H2: thyroglobulin; H2: thyroid hormone synthesis/function	Supportive thyroid-physiology evidence	—	—	—
Effects of dietary soy isoflovones on health, steroidogenesis, and thyroid gland function in dogs (2009)<br>DOI: 10.2460/ajvr.70.3.353	Animal intervention (dogs)	C	Dogs	H2: thyroid hormone synthesis/function	Supportive thyroid-physiology evidence	—	Human RCT	A
Short-term effect of soy consumption on thyroid hormone levels and correlation with phytoestrogen level in healthy subjects. (2008)<br>DOI: —	Nonrandomized human / exposure study	B	Healthy participants / exposure study	H1: thyroid autoimmunity (TPOAb/TgAb); H2: thyroid hormone synthesis/function	Supportive thyroid-physiology evidence	—	—	—
The flavonoid quercetin regulates growth and gene expression in rat FRTL-5 thyroid cells. (2008)<br>DOI: 10.1210/en.2007-0618	Animal model	C	Rat model	H2: NIS/iodide uptake; H2: thyroid hormone synthesis/function	Supportive thyroid-physiology evidence	—	—	—
Actual levels of soy phytoestrogens in children correlate with thyroid laboratory parameters. (2006)<br>DOI: 10.1515/cclm.2006.031	Nonrandomized human / exposure study	B	Human participants / exposure study	H1: thyroid autoimmunity (TgAb); H2: thyroid hormone synthesis/function	Supportive thyroid-physiology evidence	—	—	—
Inhibition of thyroid peroxidase by Myrcia uniflora flavonoids. (2006)<br>DOI: 10.1021/tx0501684	In vitro / cell model	C	Cell/biochemical model	H2: TPO; H2: thyroid hormone synthesis/function	Supportive thyroid-physiology evidence	—	—	—
Xanthohumol stimulates iodide uptake in rat thyroid-derived FRTL-5 cells. (2005)<br>DOI: 10.1002/mnfr.200500053	Animal model	C	Rat model	H2: NIS/iodide uptake; H2: thyroid hormone synthesis/function	Supportive thyroid-physiology evidence	—	—	—
Dietary genistein inactivates rat thyroid peroxidase in vivo without an apparent hypothyroid effect. (2000)<br>DOI: 10.1006/taap.2000.9019	Animal model	C	Rat model with hypothyroidism	H1: oxidative stress/antioxidant; H2: TPO; H2: thyroid hormone synthesis/function	Supportive thyroid-physiology evidence	—	—	—
Thyroid peroxidase inhibition by Kalanchoe brasiliensis aqueous extract. (2000)<br>DOI: 10.1016/s0278-6915(00)00017-x	In vitro / cell model	C	Cell/biochemical model	H2: TPO	Supportive thyroid-physiology evidence	—	—	—
Effects of polyphenol compounds from Alchemilla vulgaris on morphofunctional state of thyroid gland in rats exposed to low temperature (1999)<br>DOI: 10.1007/bf02433301	Animal model	C	Rat model	H2: thyroid hormone synthesis/function	Supportive thyroid-physiology evidence	—	—	—
Anti-thyroid isoflavones from soybean: isolation,	Experimental / other	D	Experimental/mechanistic	H1: oxidative stress/antioxidant;	Supportive thyroid-	—	—	—

Study / DOI	Final evidence type	Final level	Population / model	H1/H2 relevance	Final evidence role	Linked study cluster	Original evidence type (audit only)	Original level (audit only)
characterization, and mechanisms of action. (1997) DOI: 10.1016/s0006-2952(97)00301-8			model	H2: TPO; H2: thyroglobulin; H2: thyroid hormone synthesis/function	physiology evidence			
Flavonoids extracted from fonio millet (Digitaria exilis) reveal potent antithyroid properties. (1996) DOI: 10.1016/0899-9007(96)90707-8	Experimental / other	D	Experimental/mechanistic model	H2: TPO; H2: thyroid hormone synthesis/function	Supportive thyroid-physiology evidence	—	—	—
Inhibition of thyroid peroxidase by dietary flavonoids. (1996) DOI: 10.1021/tx950076m	Experimental / other	D	Experimental/mechanistic model	H1: oxidative stress/antioxidant; H2: TPO	Supportive thyroid-physiology evidence	—	—	—
In vivo effects of flavonoid EMD 21388 on thyroid hormone secretion and metabolism in rats. (1991) DOI: 10.1152/ajpendo.1991.261.2.e227	Animal model	C	Rat model	H2: NIS/iodide uptake; H2: thyroid hormone synthesis/function	Supportive thyroid-physiology evidence	—	—	—

Panel C. Contextual evidence

Study / DOI	Final evidence type	Final level	Population / model	H1/H2 relevance	Final evidence role	Linked study cluster	Original evidence type (audit only)	Original level (audit only)
Effects of plant flavonoids and selenium on pancreatic hormones and glucose homeostasis in autoimmune thyroiditis. (2026) DOI: 10.1538/expanim.26-0007	Animal model	C	Rat model with autoimmune thyroiditis	Indirect metabolic context in AIT; no direct H1/H2 thyroid-specific endpoint used for core interpretation	Contextual evidence	—	Experimental / other	D
Therapeutic potential of five frequently prescribed herbs in obesity-associated Hashimoto's thyroiditis: insights from efferocytosis regulation. (2025) DOI: 10.3389/fmed.2025.1538867	Computational / network pharmacology	D	Computational/network pharmacology model	H1: inflammation/immunomodulation; H2: thyroid hormone synthesis/function	Contextual evidence	—	Experimental / other	D
Achievements in Hypothyroidism Treatment with Herbal Medicine: A Systematic Review of Randomized Controlled Trials. (2023) DOI: 10.2174/1570163820666230403091841	Systematic review	D	Published human/interventional studies	Secondary synthesis: thyroid function and thyroid-autoimmunity outcomes	Contextual evidence	—	Human RCT	A
Do Dietary Supplements Affect Inflammation, Oxidative Stress, and Antioxidant Status in Adults with Hypothyroidism or Hashimoto's Disease?-A Systematic Review of Controlled Trials. (2023) DOI: 10.3390/antiox12101798	Systematic review	D	Published human/interventional studies	H1: oxidative stress/antioxidant; H1: inflammation/immunomodulation	Contextual evidence	—	—	—
The Influence of Nutritional Intervention in the Treatment of Hashimoto's Thyroiditis-A Systematic Review. (2023) DOI: 10.3390/nu15041041	Systematic review	D	Published human/interventional studies	H1: inflammation/immunomodulation; H2: thyroid hormone synthesis/function	Contextual evidence	—	—	—

Notes. HT, Hashimoto's thyroiditis; AIT, autoimmune thyroiditis; H1, immune/redox axis; H2, direct thyroid-physiology axis; TPO, thyroid peroxidase; NIS, sodium-iodide symporter. Evidence-role definitions follow the revised manuscript: core HT/AIT evidence directly investigates HT or experimental AIT; supportive thyroid-physiology evidence consists of primary studies outside HT/AIT that directly interrogate thyroid-specific H2 processes; contextual evidence provides secondary or indirect mechanistic, metabolic, pharmacological, toxicological, pharmacokinetic, exposure-related, or computational context and is not treated as independent evidence of therapeutic efficacy. Evidence levels A-D reproduce the project classification scheme. Classification corrections were performed separately from screening/adjudication decisions and did not change study eligibility; they changed the evidentiary weighting used in the final interpretation. TPOAb, thyroid peroxidase autoantibody; TgAb, thyroglobulin autoantibody. Direct H2 labels for TPO and thyroglobulin do not refer to these circulating autoantibodies.
